# Momentary Manifestations of Negative Symptoms as Predictors of Clinical Outcomes in People at High Risk for Psychosis: Experience Sampling Study

**DOI:** 10.2196/30309

**Published:** 2021-11-19

**Authors:** Isabell Paetzold, Karlijn S F M Hermans, Anita Schick, Barnaby Nelson, Eva Velthorst, Frederike Schirmbeck, EU-GEI High Risk Study, Jim van Os, Craig Morgan, Mark van der Gaag, Lieuwe de Haan, Lucia Valmaggia, Philip McGuire, Matthew Kempton, Inez Myin-Germeys, Ulrich Reininghaus

**Affiliations:** 1 Department of Public Mental Health, Central Institute of Mental Health Medical Faculty Mannheim Heidelberg University Mannheim Germany; 2 Department of Neuroscience Center for Contextual Psychiatry KU Leuven Leuven Belgium; 3 Orygen Parkville, Victoria Australia; 4 Centre for Youth Mental Health The University of Melbourne Parkville, Victoria Australia; 5 Department of Psychiatry Icahn School of Medicine at Mount Sinai New York, NY United States; 6 Department of Psychiatry Amsterdam UMC, Location AMC University of Amsterdam Amsterdam Netherlands; 7 Arkin Institute for Mental Health Amsterdam Netherlands; 8 See Acknowledgments Maastricht Netherlands; 9 Department of Psychiatry and Neuropsychology School for Mental Health and Neuroscience Faculty of Health, Medicine and Life Sciences, Maastricht University Maastricht Netherlands; 10 Department of Psychosis Studies Institute of Psychiatry King’s College London London United Kingdom; 11 Department of Psychiatry Brain Center Rudolf Magnus Utrecht University Medical Centre Utrecht Netherlands; 12 ESRC Centre for Society and Mental Health King's College London London United Kingdom; 13 Department of Health Service and Population Research Centre for Epidemiology and Public Health, Institute of Psychiatry, Psychology & Neuroscience School of Mental Health & Psychological Sciences, King's College London London United Kingdom; 14 Department of Clinical, Neuro and Developmental Psychology Vrije Universiteit Amsterdam Netherlands; 15 Department of Psychosis Research Parnassia Psychiatric Institute The Hague Netherlands; 16 Department of Early Psychosis Amsterdam UMC, Location AMC University of Amsterdam Amsterdam Netherlands; 17 Department of Psychology Institute of Psychiatry, Psychology and Neuroscience King's College London London United Kingdom; 18 NIHR Biomedical Research Centre, South London and Maudsley NHS Foundation Trust, London London United Kingdom

**Keywords:** ecological momentary assessment, psychotic disorder, psychopathology

## Abstract

**Background:**

Negative symptoms occur in individuals at ultrahigh risk (UHR) for psychosis. Although there is evidence that observer ratings of negative symptoms are associated with level of functioning, the predictive value of subjective experience in daily life for individuals at UHR has not been studied yet.

**Objective:**

This study therefore aims to investigate the predictive value of momentary manifestations of negative symptoms for clinical outcomes in individuals at UHR.

**Methods:**

Experience sampling methodology was used to measure momentary manifestations of negative symptoms (blunted affective experience, lack of social drive, anhedonia, and social anhedonia) in the daily lives of 79 individuals at UHR. Clinical outcomes (level of functioning, illness severity, UHR status, and transition status) were assessed at baseline and at 1- and 2-year follow-ups.

**Results:**

Lack of social drive, operationalized as greater experienced pleasantness of being alone, was associated with poorer functioning at the 2-year follow-up (*b*=−4.62, *P*=.01). Higher levels of anhedonia were associated with poorer functioning at the 1-year follow-up (*b*=5.61, *P*=.02). Higher levels of social anhedonia were associated with poorer functioning (eg, disability subscale: *b*=6.36, *P*=.006) and greater illness severity (*b*=−0.38, *P*=.045) at the 1-year follow-up. In exploratory analyses, there was evidence that individuals with greater variability of positive affect (used as a measure of blunted affective experience) experienced a shorter time to remission from UHR status at follow-up (hazard ratio=4.93, *P*=.005).

**Conclusions:**

Targeting negative symptoms in individuals at UHR may help to predict clinical outcomes and may be a promising target for interventions in the early stages of psychosis.

## Introduction

### Background

Negative symptoms occur in individuals at ultrahigh risk (UHR, also known as clinical high risk) for psychosis and have been reported to be associated with reduced quality of life and impaired functioning in cross-sectional and longitudinal studies [1-4]. Recently, several studies have demonstrated the predictive value of negative symptoms for social aspects of functioning [5,6]. Furthermore, negative symptoms have been found to be predictive of transition to psychotic disorder in UHR samples [7-12].

To date, clinical outcomes in UHR studies have primarily focused on transition to psychosis. Given that most individuals at UHR do not develop psychosis (71%-76%) as indicated in meta-analyses and systematic reviews [13-16], investigating other outcomes has received increasing attention in recent years [17,18]. Meta-analyses have found that most individuals at UHR who do not transition to psychosis do not remit from UHR status within 2 years either [19]. In addition, individuals at UHR—regardless of whether they transition to psychosis—show other clinical symptoms and marked impairments in functioning that are comparable with those reported in patients with social phobia and major depressive disorder [18-23]. The level of functioning in individuals at UHR is more similar to that which is observed in patients with psychotic disorders than in controls [20]. Hence, level of functioning and persistence of clinical symptoms are important outcomes other than transition to psychosis.

Standard measures used to assess negative symptoms (eg, the Positive and Negative Syndrome Scale) [24,25], though valid in their own right, have been criticized for being overly reliant on behavioral observation and third-party anamnesis [26-28]. In addition, standardized self-report questionnaires and laboratory measures of negative symptoms in patients with psychosis do not seem to converge with real-time and real-world reports generated using the experience sampling methodology (ESM) and hence may capture different constructs [29,30]. ESM is a semistructured diary method that captures daily behavior and experience of company with high ecological validity [31]. A recent systematic review of experience sampling studies investigating everyday social experiences of individuals with schizophrenia [32] concluded that, compared with other methods, experience sampling allows a more granular assessment of social experience. This underscores the importance of examining the perspective of individuals’ experience of negative symptoms in daily life (ie, momentary manifestations of negative symptoms), as this is when psychiatric symptoms naturally emerge. Experience sampling studies have made important contributions to our understanding of psychosis, but until now, studies of momentary experience of social context and manifestations of negative symptoms have mainly focused on individuals with a psychotic disorder [26,33].

Previous experience sampling studies have investigated blunted affective experience, lack of social drive, anhedonia, and social anhedonia as momentary manifestations of negative symptoms in daily life. Blunted affective experience has been operationalized as intensity (ie, mean level), instability (ie, differences in affect from one moment to the following), and variability (ie, differences between affect in the moment and the average individual affect) of positive and negative affect [26,33-35]. Lack of social drive has been assessed using the amount of time spent alone, the preference to be alone when in company, and the experienced pleasantness of being alone [35,36]. Anhedonia has been operationalized as a smaller increase of positive affect in moments of pleasant events [26,35]. Similarly, social anhedonia has been operationalized as a smaller increase in positive affect associated with being in pleasant company [26,35,36].

To our knowledge, only 2 experience sampling studies have, to date, investigated momentary manifestations of negative symptoms in individuals at UHR [35,37]. Although differing in focus and operationalization of constructs, both studies compared momentary manifestations of negative symptoms across individuals at UHR, patients with first-episode psychosis, and controls. In line with findings in enduring psychosis [26,38], both studies concluded that there may be a mismatch between what individuals at UHR experience and how they express this in their behavior, that may be interpreted as 2 distinct dimensions of negative symptoms (ie, experience vs expression). Hence, assessing individuals’ subjective experience of negative symptoms is important to gain a more comprehensive understanding of internal, experiential aspects [27]. However, both studies used a cross-sectional design. No experience sampling study to date has used momentary manifestations of negative symptoms for predicting clinical outcomes in individuals at UHR in a longitudinal design. This is an important gap that needs to be addressed, as a shift in research toward subjective experience of momentary symptoms may offer new insights into the social nature and development of negative symptoms in UHR and its outcomes.

### Objectives

This study aims to investigate whether momentary manifestations of negative symptoms predict clinical outcomes (ie, illness severity, level of functioning, and remission from UHR status and transition to psychosis) in individuals at UHR for psychosis at the 1- and 2-year follow-ups. We tested the following hypotheses:

Momentary manifestations of negative symptoms in daily life predict clinical outcomes in individuals at UHR at 1- and 2-year follow-up such that higher levels of (1) blunted affective experience (ie, lower intensity, variability and instability of positive and negative affect; H1, hypothesis 1); (2) lack of social drive (ie, amount of time spent alone, pleasantness of being alone, and preference to be alone when in company; H2); (3) anhedonia (ie, no or low increase of positive affect in moments of pleasant events; H3); and (4) social anhedonia (ie, no or low increase of positive affect in moments of pleasant company; H4) are associated with greater illness severity and poorer functioning at follow-up.

In exploratory analyses, we further aimed to examine whether momentary manifestations of negative symptoms are associated with time to transition to psychosis or remission from UHR status.

## Methods

### Sample

We recruited a sample of individuals at UHR aged 15-35 years, who were assessed at baseline and 1- and 2-year follow-up. Participants were recruited in London (United Kingdom), Melbourne (Australia), and Amsterdam and The Hague (the Netherlands) as a part of the high-risk study of the European Network of National Schizophrenia Networks Studying Gene-Environment Interactions (EU-GEI [39]). EU-GEI is a naturalistic prospective multicenter study that aims to identify the interactive genetic, clinical, and environmental determinants of schizophrenia.

To be eligible to participate, individuals had to meet at least one of the UHR criteria as defined by the Comprehensive Assessment of At Risk Mental States [40]: (1) attenuated psychotic symptoms: the presence of subthreshold positive psychotic symptoms for at least 1 month during the past year; (2) brief limited intermittent psychotic symptoms: an episode of frank psychotic symptoms that lasted no longer than 1 week, which abated spontaneously; or (3) vulnerability: a first-degree relative with a psychotic disorder or schizotypal personality disorder in combination with a significant drop in functioning during at least 1 month in the previous year or enduring low functioning. Exclusion criteria were (1) presence of a current or past psychotic disorder; (2) symptoms for inclusion explained by a medical disorder, drugs or alcohol dependency; or (3) intelligence quotient<60.

### Data Collection

#### Experience Sampling Measures

Data on momentary manifestations of negative symptoms were collected using ESM [31,41]. Participants were asked to report their thoughts, feelings, and symptoms as well as the context (eg, location, company, activity) and the appraisal of the context in their normal daily lives [41-44]. For data collection, participants used a dedicated digital device (the Psymate), which prompted participants with a *beep* to complete a brief questionnaire 10 times a day on 6 consecutive days at random moments within set blocks of time.

A detailed description of ESM items and compliance procedure is provided in Table 1. Momentary manifestations of negative symptoms were operationalized as follows: for blunted affective experiences, we computed mean levels of intensity, variability, and instability of positive and negative affect across beeps within participants. We used 3 operationalizations for lack of social drive: the amount of time spent alone as the percentage of total time, the preference for being alone when in company, and the pleasantness of being alone. To represent anhedonia, we obtained fitted values of positive affect predicted by event pleasantness. As anhedonia is by definition related to pleasant events, only ratings of 1 to 3 were used to test associations with positive affect [26,35]. Observations that indicated unpleasant events (−3 to −1) were excluded from analysis, and neutral events (0) were set as the reference category [26]. We fitted a 2-level, linear mixed model with pleasantness of being in company as the independent and positive affect as the outcome variable and obtained fitted values for representing social anhedonia.

Consistent with previous research, psychometric properties for measures of momentary manifestations of negative symptoms were assessed by evaluating their convergent validity. Therefore, we examined the association between momentary manifestations and observer-rated measures of negative symptoms at baseline (assessed with the expanded Brief Psychiatric Rating Scale [BPRS] [45]; Multimedia Appendix 1). We found small to moderate correlations between the BPRS total score and intensity of negative (*r*=0.28, *P*=.02) and positive affect (*r*=−0.34, *P*=.004), variability of negative affect (*r*=0.26, *P*=.03), anhedonia (*r*=−0.34, *P*=.003), and social anhedonia (*r*=−0.31, *P*=.008). We found no evidence that the BPRS negative symptom subscale was associated with momentary manifestations of negative symptoms. In addition, we used observer-rated measures of negative symptoms to predict momentary manifestations of negative symptoms in a multilevel model (Multimedia Appendix 1). BPRS total score predicted intensity of positive (b=0.04, *P*=.01) and negative affect (b=−0.04, *P*<.001), instability (b=0.04, *P*=.03) and variability (b=0.03, *P*=.003) of negative affect, anhedonia (b=−0.04, *P*=.001), and social anhedonia (b=−0.04, *P*=.001). The BPRS negative symptoms scale did not predict momentary manifestations of negative symptoms in the multilevel model.

#### Clinical Outcomes

Clinical outcomes were assessed at baseline, and approximately 1 and 2 years after the baseline assessment. As participants were not seen at exactly 1 and 2 years from their baseline appointment, the exact time points for follow-up assessments varied. Hence, the data closest to 1 and 2 years after baseline were selected as follow-up data. Transition to psychosis and UHR status were assessed using the Comprehensive Assessment of At Risk Mental States [40]. If participants could not be reinterviewed for the follow-up assessments, clinical notes were used to determine transition status. Participants’ level of functioning was assessed using the symptoms and the functioning subscales of the Global Assessment of Functioning (GAF [46]) scale. Illness severity was assessed using the severity of illness subscale of the Clinical Global Impression [47] scale. A detailed description of the outcome measures is provided in Table 1.

**Table 1:** Overview of experience sampling and clinical outcome measures.

Domain		Measure
**Experience sampling^a^**		
	Positive affect	Positive affect was measured by asking participants to rate how cheerful, relaxed, satisfied, and enthusiastic they felt on a Likert scale ranging from 1 (not at all) to 7 (very much). We found satisfying internal consistency, Cronbach *α*=.73. In line with previous studies [34,48], we used high and low physiological arousal items.
	Negative affect	Negative affect was measured by asking participants to rate the extent to which they felt insecure, down, lonely, anxious and irritated on a Likert scale ranging from 1 (not at all) to 7 (very much). We found satisfying internal consistency, Cronbach *α*=.73.
	Blunted affect	Intensity was operationalized as the mean levels of positive and negative affect. Instability was computed as the squared difference between beep-level positive and negative affect intensity at beep t and beep-level positive affect intensity at beep t-1 (previous beep), within days, within persons (mean of the squared successive differences), and only calculated if there was a maximum of 2 observations missing between 2 consecutive observations. Difference scores between 2 observations overnight were excluded [33]. Variability was computed as the squared difference between beep-level intensity of positive and negative affect at each observation and individual mean positive and negative affect over observations, over days within persons [33].
	Social drive	Lack of social drive was conceptualized as the amount of time spent alone in percentage of total time, the experienced pleasantness of being alone, and the preference of being alone when in company. Pleasantness of being alone and preference to be alone when in company were rated on a Likert scale ranging from 1 (not at all) to 7 (very much). If participants were alone: “I find it pleasant to be alone” and “I would prefer to have company.” If participants were in company: “I find being with these people pleasant.” and “I would prefer to be alone.”
	Anhedonia	Anhedonia was conceptualized as the relationship between positive affect and the occurrence of pleasant events. Participants were asked to think about the most important event that happened since the last beep. The pleasantness of this event was rated on a bipolar scale ranging from −3 (very unpleasant) to 3 (very pleasant). We only used ratings of 1 to 3 to test associations with positive affect, as anhedonia is per definition related to pleasant events. Observations indicating unpleasant events (−3 to −1) were excluded, and neutral events (0) were used as a reference category [26].
	Social anhedonia	Social anhedonia was defined as the association between positive affect and pleasantness of being in company [26]. Participants were asked whether they were alone or in company. If participants indicated to be in company, they were asked to rate “I find being with these people pleasant.” on a Likert scale ranging from 1 (not at all) to 7 (very much).
**Clinical outcome measures**		
	CAARMS	Transition to psychosis and UHR status were assessed using the Comprehensive Assessment of At Risk Mental State (CAARMS [40]), a semistructured interview to assess attenuated psychotic symptoms in individuals at high risk for psychosis. The CAARMS comprises 27 items clustered in 7 subscales: positive symptoms, cognitive change (attention and concentration), emotional disturbance, negative symptoms, behavioral change, motor or physical changes, and general psychopathology. Scores on each item range from 0 (absent) to 6 (extreme).
	GAF	The Global Assessment of Functioning (GAF [46]) obtains ratings of burdening symptoms and disabilities in the last month on a scale from 100 (no symptoms or superior functioning in a wide range of activities) to 1 (persistent danger of severely hurting self or others or serious suicidal act with clear expectation of death or persistent inability to maintain minimal personal hygiene).
	CGI	The Clinical Global Impression Scale (CGI [47]) symptoms severity subscale is an expert rating of average illness severity during the last week ranging from 1 (normal, not at all ill) to 7 (among the most extremely ill patients).

^a^Experience sampling procedure: During an initial briefing, the study team ensured that the week of data collection was a typical week for the participant. Each time the device emitted the beep signal, participants were asked to stop their activity and answer the questions. The experience sampling questionnaire was available to participants for the duration of 10 minutes after emission of the beep signal. Participants were contacted at least once during the assessment period to assess their adherence to instructions, identify any potential distress associated with the method, and maximize the number of observations per participant. At the end of the assessment period, participants’ reactivity to, and compliance with, the method was examined in a debriefing session. Participants were required to provide valid responses to at least one-third (ie, 20 valid answers) of the emitted beeps to be included in the analysis [49]. Procedures to ensure data quality are reported in Multimedia Appendix 2.

### Statistical Analysis

Momentary manifestations of negative symptoms—operationalized and computed as detailed above—were used as independent variables to predict clinical outcomes at 1- and 2-year follow-up using Stata 15. We fitted linear regression models using the command *regress* with level of functioning and illness severity as outcome variables and momentary manifestations of negative symptoms as independent variables. In exploratory analyses, we examined the predictive value of momentary manifestations of negative symptoms for transition to psychosis and remission from UHR status as outcomes. Survival analyses using the Stata commands *stset* and *streg* were performed to account for the time to event structure of the data. We used time to follow-up as a proxy for time to remission. In both survival analyses, a Weibull distribution was assumed.

Analyses were adjusted for a priori confounders (ie, age, gender, ethnicity, center, time to follow-up; unadjusted results are provided in Multimedia Appendix 3). In a sensitivity analysis, we included current depressive episode (Multimedia Appendix 4) and comorbid disorders (Multimedia Appendix 5) as additional independent variables to control for potential confounding. We corrected for multiple testing to reduce the probability of type I errors because of the number of tests performed. We corrected within domains of momentary manifestations of negative symptoms and clinical outcomes. As in previous experience sampling studies [50,51], Simes correction method was used to account for multiple tests of significance [52]. Simes correction is a modified version of the more conservative Bonferroni correction in case of dependent hypotheses given significance tests in the current analyses were not independent [52]. With the Simes correction, the most significant *P* value is tested against α=.05/n (total number of tests), the second most significant *P* value is tested against α=.05/(n−1), the third *P* value against α=.05/(n−2), and so on. Simes-corrected significant results are highlighted in a footnote in tables.

## Results

### Basic Sample and Clinical Characteristics

The ESM sample comprised 79 individuals at UHR, of whom 9 transitioned to psychosis during the study period. Data on clinical outcomes were obtained for 48 individuals at 1-year follow-up and 36 individuals at 2-year follow-up. Participants were on average aged 23 (SD 4.93) years and 56% (44/79) were women. The majority (53/79, 67%) of the sample was White, followed by 15% (12/79) with Black ethnicity. In addition to their UHR status, 76% (60/79) of the participants were diagnosed with a comorbid axis I disorder (further details are provided in Multimedia Appendix 6). Compared with the sample of individuals included in the EU-GEI High Risk Study for whom experience sampling data were not collected (no ESM sample, N=266), samples in this study showed no differences in demographic characteristics (age: t_343_=−1.33, *P*=.19; gender: *χ*^2^_1_=3.6, *P*=.06; ethnicity: *χ*^2^_5_=6.5, *P*=.26) or prevalence of comorbid disorders (*χ*^2^_1_=1.8, *P*=.18). However, the current sample showed poorer functioning (symptoms: t_315_=2.29, *P*=.02) and lower levels of observer-rated negative symptoms (t_320_=2.27, *P*=.02) at baseline. Comparing participants who completed follow-up assessments, the sample with no ESM data collected (N=134, 1-year follow-up; N=89, 2-year follow-up) showed a lower BPRS total score at 1-year follow-up (t_159_=−2.07, *P*=.04). There were no significant differences in demographic or clinical characteristics at 2-year follow-up. Table 2 gives an overview of relevant sample characteristics.

**Table 2:** Basic sample and clinical characteristics.^a^

Characteristics			ESM^b^ sample							No ESM sample							ESM vs no ESM						
			Baseline		1-year follow-up		2-year follow-up		Baseline			1-year follow-up		2-year follow-up		Baseline			1-year follow-up			2-year follow-up	
Sample size			79		48		36		266			134		89		N/A^c^			N/A			N/A	
Age at baseline (years), mean (SD)			23.0 (4.93)		23.6 (5.24)		23.8 (5.18)		22.2 (4.89)			22.5 (4.82)		23.3 (5.14)		*t*_343_=−1.33, *P*=.19			*t*_180_=−1.30, *P*=.19			*t*_123_=−.45, *P=*.65	
**Gender, n (%)**																	*χ*^2^_1_=3.58, *P*=.06			*χ*^2^_1_=3.76, *P*=.05			*χ*^2^_1_=2.74, *P*=.10
	Male	35 (44)		22 (46)		16 (44)		150 (56)			83 (62)		54 (61)										
	Female	44 (56)		26 (54)		20 (56)		116 (44)			51 (38)		35 (39)										
**Ethnicity, n (%)**																	*χ*^2^_5_=6.5, *P*=.26			*χ*^2^_5_=6.7, *P*=.24			*χ*^2^_5_=6.25, *P*=.28
	White	53 (67)		33 (69)		27 (75)		193 (73)			99 (74)		63 (71)										
	Black	12 (15)		9 (19)		5 (14)		22 (8)			9 (7)		6 (7)										
	Other	14 (18)		6 (13)		4 (11)		50 (19)			26 (19)		20 (22)										
Comorbidity at baseline, n(%)			60 (76)		37 (77)		28 (78)		220 (83)			111 (83)		79 (89)		*χ*^2^_1_=1.8, *P*=.18			*χ*^2^_1_=0.77, *P*=.38			*χ*^2^_1_=2.5, *P*=.11	
**BPRS^d^**																							
	Total score, mean (SD)	44.00 (9.46)		39.69 (11.63)		37.31 (12.53)		43.37 (10.57)			36.01 (9.70)		33.45 (10.85)		*t*_314_=−0.46, *P*=.65			*t*_159_=−2.07, *P*=.04			*t*_111_=−1.97, *P*=.05		
	Negative symptom score, mean (SD)	4.49 (1.86)		4.04 (1.74)		3.75 (1.78)		5.21(2.51)			4.42 (2.05)		4.12(1.87)		*t*_320_=2.27, *P*=.02			*t*_160_=1.22, *P*=.26			*t*_112_=−.98, *P*=.33		
**GAF^e^**																							
	Symptoms, mean (SD)	52.88 (9.85)		56.96 (10.76)		61.00 (11.73)		55.92 (10.23)			59.49 (13.08)		61.25 (15.02)		*t*_315_=2.29, *P*=.02			*t*_180_=1.20, *P*=.23			*t*_123_=0.09, *P*=.93		
	Disability, mean (SD)	56.27 (13.00)		58.92 (13.41)		63.78 (13.62)		55.36 (12.20)			60.40 (13.77)		61.81 (16.09)		*t*_330_=−0.57, *P*=.57			*t*_196_=0.65, *P*=.51			*t*_132_=−0.65, *P*=.51		
**CGI^f^**																							
	Illness severity, mean (SD)	3.57 (1.21)		3.15 (1.32)		2.89 (1.25)		3.60 (1.09)			3.33 (1.37)		3.22 (1.51)		*t*_319_=0.21, *P*=.83			*t*_203_=0.83, *P*=.41			*t*_148_=1.21, *P*=.23		
UHR^g^ criteria met, n (%)			N/A		36 (73)		23 (62)		N/A			107 (73)		71 (66)		N/A			*χ*^2^_1_=.00, *P*=.97			*χ*^2^_1_=.15, *P*=.69	

^a^Follow-up values for age, gender, ethnicity, and comorbidity based on individuals with valid Global Assessment of Functioning Scale at follow-up.

^b^ESM: Experience Sampling Methodology.

^c^N/A: not applicable.

^d^BPRS: Brief Psychiatric Rating Scale.

^e^GAF: Global Assessment of Functioning Scale.

^f^CGI: Clinical Global Impression Scale.

^g^UHR: ultrahigh risk.

### Blunted Affective Experience and Clinical Outcomes

Tables 3 and 4 show the results on clinical outcomes at follow-up predicted by blunted affective experience at baseline. We found no evidence that blunted affective experience predicted illness severity or level of functioning. In exploratory analyses, time to remission from UHR status was predicted by variability of positive affect (hazard ratio [HR]=4.93, 95% CI 1.61-15.11, *P*=.005, statistically significant after Simes correction). Participants with greater variability were more likely to experience a shorter time to remission from the UHR status. We found no evidence that blunted affective experience predicted transition to psychosis.

**Table 3:** Level of functioning at 1- and 2-year follow-up predicted by blunted affective experience at baseline (ie, intensity, instability, and variability of negative and positive affect) and clinical outcome at baseline.^a^

Outcomes		Level of functioning								
		Symptoms^b^				Disability^b^				
		1-year follow-up (n=48)		2-year follow-up (n=36)		1-year follow-up (n=48)			2-year follow-up (n=36)	
		*b* (CI)	*P* value	*b* (CI)	*P* value	*b* (CI)	*P* value	*b* (CI)		*P* value
**Predictor: Intensity NA^c^**										
	Outcome at baseline	0.18 (−0.16 to 0.52)	.29	0.00 (−0.61 to 0.61)	.99	0.34 (−0.01 to 0.70)	.06	0.55 (0.06 to 1.04)		.03
	Intensity NA	−2.51 (−6.54 to 1.52)	.22	−1.36 (−6.89 to 4.18)	.62	−3.17 (−7.83 to 1.48)	.18	1.26 (−4.90 to 7.42)		.68
**Predictor: Intensity PA^d^**										
	Outcome at baseline	0.15 (−0.18 to 0.48)	.36	−0.01 (−0.62 to 0.60)	.97	0.34 (0.00 to 0.68)	.05	0.55 (0.06 to 1.05)		.03
	Intensity PA	3.84 (−0.09 to 7.77)	.06	1.15 (−4.58 to 6.88)	.68	5.04 (0.59 to 9.49)	.03	0.07 (−6.34 to 6.48)		.98
**Predictor: Instability NA**										
	Outcome at baseline	0.24 (−0.10 to 0.58)	.17	−0.02 (−0.62 to 0.58)	.94	0.36 (0.00 to 0.73)	.05	0.55 (0.08 to 1.01)		.02
	Instability NA	0.80 (−1.43 to 3.04)	.47	−1.72 (−5.94 to 2.50)	.41	−0.36 (−2.98 to 2.27)	.79	−3.66 (−8.21 to 0.88)		.11
**Predictor: Instability PA**										
	Outcome at baseline	0.22 (−0.12 to 0.56)	.21	−0.06 (−0.64 to 0.52)	.83	0.37 (0.01 to 0.73)	.046	0.55 (0.11 to 0.99)		.02
	Instability PA	−0.24 (−3.75 to 3.28)	.89	−4.68 (−10.43 to 1.07)	.11	−0.46 (−4.57 to 3.64)	.82	−7.52 (−13.54 to −1.51)		.02
**Predictor: Variability NA**										
	Outcome at baseline	0.23 (−0.11 to 0.56)	.18	0.00 (−0.59 to 0.60)	.99	0.37 (0.01 to 0.73)	.046	0.52 (0.07 to 0.98)		.03
	Variability NA	1.60 (−3.30 to 6.50)	.51	−3.80 (−11.52 to 3.92)	.32	0.03 (−5.76 to 5.82)	.99	−7.96 (−16.15 to 0.22)		.06
**Predictor: Variability PA**										
	Outcome at baseline	0.21 (−0.13 to 0.55)	.22	0.02 (−0.56 to 0.60)	.93	0.37 (0.01 to 0.73)	.04	0.47 (−0.01 to 0.95)		.05
	Variability PA	1.12 (−4.54 to 6.77)	.6.92	−5.55 (−12.51 to 1.42)	.11	2.22 (−4.34 to 8.78)	.50	−6.30 (−14.23 to 1.63)		.11

^a^Results adjusted for age, gender, ethnicity, center, and time to follow-up.

^b^Level of functioning assessed with the Global Assessment of Functioning Scale.

^c^NA: negative affect.

^d^PA: positive affect.

**Table 4:** Illness severity, remission from ultrahigh risk (UHR) status and transition status 1- and 2-year follow-up predicted by blunted affective experience at baseline (ie, intensity, instability, and variability of negative and positive affect) and clinical outcome at baseline.^a^

Outcomes			Illness severity^b^									Remission from UHR status					Transition status		
			1-year follow-up (n=47)				2-year follow-up (n=37)												
			*b* (CI)		*P* value		*b* (CI)		*P* value		HR^c^ (CI)			*P* value		HR (CI)			*P* value
**Predictor: Intensity NA^d^**																			
	Outcome at baseline	0.43 (0.13 to 0.73)		.006		0.28 (−0.19 to 0.75)		.24		N/A^e^			N/A		N/A			N/A	
	Intensity NA	0.32 (−0.07 to 0.71)		.11		−0.03 (−0.59 to 0.53)		.91		0.34 (0.12 to 0.98)			.045		1.44 (0.66 to 3.13)			.36	
**Predictor: Intensity PA^f^**																			
	Outcome at baseline	0.44 (0.15 to 0.74)		.004		0.17 (−0.30 to 0.64)		.46		N/A			N/A		N/A			N/A	
	Intensity PA	−0.31 (−0.69 to 0.07)		.11		−0.35 (−0.98 to 0.28)		.26		2.08 (0.88 to 4.93)			.10		0.62 (0.23 to 1.65)			.34	
**Predictor: Instability NA**																			
	Outcome at baseline	0.52 (0.22 to 0.81)		.001		0.27 (−0.18 to 0.72)		.23		N/A			N/A		N/A			N/A	
	Instability NA	−0.02 (−0.23 to 0.18)		.81		−0.02 (−0.46 to 0.42)		.94		1.19 (0.57 to 2.48)			.64		1.02 (0.67 to 1.54)			.92	
**Predictor: Instability PA**																			
	Outcome at baseline	0.51 (0.22 to 0.81)		.001		0.28 (−0.16 to 0.73)		.20		N/A			N/A		N/A			N/A	
	Instability PA	−0.06 (−0.38 to 0.26)		.71		0.24 (−0.38 to 0.86)		.43		1.75 (1.69 to 4.44)			.24		0.99 (0.50 to 1.94)			.97	
**Predictor: Variability NA**																			
	Outcome at baseline	0.51 (0.22 to 0.81)		.001		0.26 (−0.20 to 0.72)		.25		N/A			N/A		N/A			N/A	
	Variability NA	−0.10 (−0.53 to 0.33)		.64		−0.08 (−0.89 to 0.74)		.85		1.24 (0.30 to 5.14)			.77		1.21 (0.55 to 2.63)			.64	
**Predictor: Variability PA**																			
	Outcome at baseline	0.51 (0.21 to 0.81)		.001		0.37 (−0.09 to 0.83)		.11		N/A			N/A		N/A			N/A	
	Variability PA	0.04 (−0.47 to 0.54)		.88		0.49 (−0.29 to 1.28)		.21		4.93 (1.61 to 15.11)			.005^g^		1.49 (0.52 to 4.23)			.46	

^a^Results adjusted for age, gender, ethnicity, center, and time to follow-up.

^b^Illness severity assessed with the Clinical Global Impression Scale.

^c^HR: hazard ratio.

^d^NA: negative affect.

^e^N/A: not applicable.

^f^PA: positive affect.

^g^Statistically significant after Simes correction.

### Lack of Social Drive and Clinical Outcomes

Tables 5 and 6 show findings on clinical outcomes predicted by lack of social drive. We found no evidence that the amount of time spent alone and the preference to be alone when in company predicted level of functioning or illness severity. Experienced pleasantness of being alone predicted the GAF disability subscale at 2-year follow-up (b=−4.62; 95% CI −8.19 to −1.04, *P*=.01 [statistically significant after Simes correction]), such that individuals who experienced greater pleasantness of being alone showed poorer functioning. However, there was no evidence that pleasantness of being alone predicted illness severity and the GAF symptoms score. In exploratory analyses, there was no evidence that lack of social drive predicted time to transition or remission from UHR status.

**Table 5:** Level of functioning at 1- and 2-year follow-up predicted by lack of social drive (ie, amount of time spent alone, preference to be alone when in company and experienced pleasantness of being alone) and clinical outcome at baseline.^a^

Outcomes			Level of functioning: symptoms^b^										Level of functioning: disability^b^							
			1-year follow-up (N=48)					2-year follow-up (N=36)					1-year follow-up (N=48)					2-year follow-up (N=36)		
			*b* (CI)		*P* value		*b* (CI)			*P* value		*b* (CI)			*P* value		*b* (CI)			*P* value
**Predictor: Amount of time spent alone**																				
	Outcome at baseline	0.22 (−0.12 To 0.56)		.20		−0.10 (−0.69 to 0.48)			.72		0.37 (0.01 to 0.73)			.045		0.49 (0.03 to 0.96)			.04	
	Amount of time spent alone	1.71 (−12.18 to 15.60)		.80		13.63 (−3.99 to 31.24)			.12		4.50 (−11.75 to 20.76)			.58		17.77 (−1.38 to 36.93)			.07	
**Predictor: Preference to be alone when in company**																				
	Outcome at baseline	0.20 (−0.13 to 0.54)		.23		0.04 (−0.58 to 0.67)			.89		0.37 (0.01 to 0.72)			.04		0.54 (0.05 to 1.03)			.03	
	Preference to be alone	−1.61 (−4.20 to 0.97)		.21		−1.38 (−5.41 to 2.65)			.49		−1.88 (−4.90 to 1.15)			.22		−1.12 (−5.46 to 3.23)			.60	
**Predictor: Pleasantness of being alone**																				
	Outcome at baseline	0.22 (−0.12 to 0.57)		.21		0.08 (−0.51 to 0.67)			.77		0.41 (0.04 to 0.79)			.03		0.51 (0.07 to 0.95)			.02	
	Pleasantness of being alone	0.04 (−2.88 to 2.96)		.98		−2.87 (−6.36 to 0.62)			.10		−1.38 (−4.86 to 2.10)			.43		−4.62 (−8.19 to −1.04)			.01^c^	

^a^Results adjusted for age, gender, ethnicity, center, and time to follow-up.

^b^Level of functioning assessed with the Global Assessment of Functioning Scale.

^c^Statistically significant after Simes correction.

**Table 6:** Illness severity, remission from ultrahigh risk (UHR) status and transition status at 1- and 2-year follow-up predicted by lack of social drive (ie, amount of time spent alone, preference to be alone when in company and experienced pleasantness of being alone) and clinical outcome at baseline.^a^

Outcomes			Illness severity^b^										Remission from UHR status					Transition status		
			1-year follow-up (N=47)					2-year follow-up (N=37)					(N=54)					(N=57)		
			*b* (CI)		*P* value		*b* (CI)			*P* value		HR^c^ (CI)			*P*		HR (CI)			*P* value
**Predictor: Amount of time spent alone**																				
	Outcome at baseline	0.49 (0.19 to 0.80)		.002		0.23 (−0.22 to 0.67)			.30		N/A^d^			N/A		N/A			N/A	
	Amount of time spent alone	−0.29 (−1.59 to 1.02)		.66		−1.17 (−3.00 to 0.66)			.20		3.91 (0.25 to 60.64)			.33		0.07 (0.00 to 2.07)			.13	
**Predictor: Preference to be alone when in company**																				
	Outcome at baseline	0.48 (0.18 to 0.78)		.002		0.24 (−0.20 to 0.68)			.27		N/A			N/A		N/A			N/A	
	Preference to be alone	0.11 (−0.14 to 0.36)		.37		0.23 (−0.17 to 0.63)			.24		0.97 (0.51 to 1.84)			.92		1.20 (0.65 to 2.22)			.56	
**Predictor: Pleasantness of being alone**																				
	Outcome at baseline	0.51 (0.21 to 0.81)		.002		0.32 (−0.13 to 0.77)			.15		N/A			N/A		N/A			N/A	
	Pleasantness of being alone	0.05 (−0.19 to 0.30)		.68		0.19 (−0.16 to 0.54)			.28		0.82 (0.44 to 1.54)			.54		1.39 (0.75 to 2.56)			.30	

^a^Results adjusted for age, gender, ethnicity, center, and time to follow-up.

^b^Illness severity assessed with the Clinical Global Impression Scale.

^c^HR: hazard ratio.

^d^N/A: not applicable.

### Anhedonia and Clinical Outcomes

Tables 7 and 8 show findings on clinical outcomes at 1- and 2-year follow-up predicted by anhedonia. Anhedonia predicted the GAF disability subscale at 1-year follow-up (b=5.61, 95% CI 1.08-10.15; *P*=.02 [statistically significant after Simes correction]). Lower positive affect in moments of pleasant events or, in other words, higher levels of anhedonia, were associated with poorer functioning. However, we found no evidence that anhedonia predicted functioning at 2-year follow-up. In addition, anhedonia did not predict illness severity at 1- and 2-year follow-up. In exploratory analyses, we found no evidence that anhedonia predicted time to remission or transition to psychosis.

**Table 7:** Level of functioning at 1- and 2-year follow-up predicted by Anhedonia, Social Anhedonia and clinical outcome at baseline.^a^

Outcomes	Level of functioning: symptoms^b^							Level of functioning: disability^b^				
	1-year follow-up (N=48)			2-year follow-up (N=36)			1-year follow-up (N=48)				2-year follow-up (N=36)	
	*b* (CI)	*P* value	*b* (CI)		*P* value	*b* (CI)			*P* value	*b* (CI)		*P* value
**Predictor: anhedonia**												
Outcome at baseline	0.16 (−0.17 to 0.49)	.32	−0.02 (−0.62 to 0.59)		.96	0.34 (0.00 to 0.68)			.048	0.56 (0.06 to 1.05)		.03
Anhedonia events	3.73 (−0.32 to 7.78)	.07	0.25 (−5.37 to 5.88)		.93	5.61 (1.08 to 10.15)			.02^c^	−0.43 (−6.70 to 5.85)		.89
**Predictor: social anhedonia**												
Outcome at baseline	0.17 (−0.15 to 0.49)	.28	0.01 (−0.59 to 0.61)		.97	0.33 (0.01 to 0.66)			.046	0.53 (0.04 to 1.01)		.04
Social anhedonia	4.61 0.74 to 8.48)	.02^c^	2.29 (−3.65 to 8.23)		.44	6.36 (1.97 to 10.74)			.006^c^	3.09 (−3.51 to 9.70)		.35

^a^Results adjusted for age, gender, ethnicity, center and time to follow-up.

^b^Level of functioning assessed with the Global Assessment of Functioning Scale.

^c^Statistically significant after Simes correction.

**Table 8:** Illness severity, remission from ultrahigh risk (UHR) status and transition status at 1- and 2-year follow-up predicted by Anhedonia, Social Anhedonia and clinical outcome at baseline.^a^

Outcomes	Illness severity^b^							Remission from UHR status				Transition status	
	1-year follow-up (N=47)			2-year follow-up (N=37)			(N=54)				(N=57)		
	*b* (CI)	*P* value	*b* (CI)		*P* value	HR^c^ (CI)			*P* value	HR (CI)			*P* value
**Predictor: anhedonia**													
Outcome at baseline	0.45 (0.15 to 0.75)	.004	0.19 (−0.29 to 0.66)		.42	N/A^d^			N/A	N/A			N/A
Anhedonia	−0.30 (−0.69 to 0.09)	.13	−0.30 (−0.91 to 0.32)		.34	2.02 (0.82 to 4.96)			.13	0.66 (0.23 to 1.88)			.44
**Predictor: social anhedonia**													
Outcome at baseline	0.44 (0.15 to 0.73)	.004	0.14 (−0.30 to 0.59)		.52	N/A			N/A	N/A			N/A
Social anhedonia	−0.38 (−0.74 to −0.01)	.045^e^	−0.57 (−1.18 to 0.05)		.07	2.22 (0.85 to 5.81)			.10	0.69 (0.26 to 1.80)			.45

^a^Results adjusted for age, gender, ethnicity, center and time to follow-up.

^b^Illness severity assessed with the Clinical Global Impression Scale.

^c^HR: hazard ratio.

^d^N/A: not applicable.

^e^Statistically significant after Simes correction.

### Social Anhedonia and Clinical Outcomes

As displayed in Tables 7 and 8, reduced positive affect in moments of pleasant company or, in other words, higher levels of social anhedonia at baseline were associated with higher levels of illness severity (b=−0.38; 95% CI −0.74 to 0.01; *P*=.045 [statistically significant after Simes correction]) and lower scores on both GAF subscales (symptoms: b=4.61; 95% CI 0.74 to 8.48; *P*=.02 [statistically significant after Simes correction]; disability: b=6.36; 95% CI 1.97 to 10.74; *P*=.006 [statistically significant after Simes correction]) at 1-year follow-up. However, we found no evidence that social anhedonia predicted clinical outcomes at 2-year follow-up. In exploratory analyses, we found no evidence that social anhedonia predicted time to remission or transition to psychosis.

## Discussion

### Principal Findings

Using an experience sampling design, this study found no evidence that blunted affective experience predicted functioning or illness severity at follow-up (H1). However, there was some evidence that higher experienced pleasantness of being alone was associated with poorer functioning at 2-year follow-up (H2). In addition, our results tentatively suggest that higher levels of anhedonia were associated with poorer functioning at 1-year follow-up (H3). Finally, we found robust evidence that higher levels of social anhedonia were associated with higher levels of illness severity and poorer functioning at 1-year follow-up (H4). In our exploratory analysis, we found no evidence that momentary manifestations of negative symptoms in daily life predicted transition status. However, our results tentatively suggest that blunted affective experience predicted time to remission from UHR status.

### Methodological Considerations

Our findings should be interpreted in light of several methodological considerations. First, the sample selection should be critically evaluated: ESM is a burdensome research method, which may lead to selection bias, such that individuals with more intense symptoms might be underrepresented in the sample. However, compared with the no ESM sample of the EU-GEI High Risk Study, the participants in this showed comparable levels of illness severity and lower scores on the GAF symptoms subscale at baseline. In addition, the sample showed high comorbidity rates of nonpsychotic disorders, which replicates findings from previous studies and systematic reviews [53,54]. High rates of comorbidity, especially comorbid depressive disorders, may have attenuated the observed effects. However, when controlling for current depressive episodes or comorbid disorders in our sensitivity analysis, we found a similar pattern in terms of magnitude of associations but slightly wider 95% CIs and some differences in statistical significance. In addition, it is important to consider the small-to-moderate sample size and the small absolute number of 9 individuals (11% of the sample) who transitioned to psychosis within the follow-up period, although this transition rate is rather common in the field [14,55]. Second, measuring social isolation and affect repeatedly over longer periods might provide a better prediction of outcomes. However, given burden on participants, this would require a less intense longitudinal data collection method, as is the case for ESM. Third, it is important to consider some limitations regarding data collection at follow-up: Although this was planned for 1- and 2-year follow-up, follow-up intervals varied in some individuals. Yet, analyses were controlled for time to follow-up and sensitivity analyses conducted with the subsample of individuals assessed ±6 months to the ideal follow-up time point showed a similar pattern of findings though varying statistical significance due to reduced sample size (Multimedia Appendix 7). Moreover, experience sampling data was not collected at follow-up. Nonetheless, using the Clinical Global Impression scale and the GAF scale, we obtained ratings of several widely used outcome measures at follow-up. In addition, the follow-up period of 2 years was, arguably, rather short in this study. However, previous research has demonstrated that the highest risk for transition in UHR samples is over the first 2 years after ascertainment [56]. Fourth, one should consider some statistical issues: For anhedonia and social anhedonia, we used fitted values of positive affect predicted by event pleasantness or pleasantness of social contact, to predict, in turn, clinical outcomes at follow-up. For blunted affective experience and lack of social drive, we aggregated data on the person-level. Aggregation of momentary manifestations of negative symptoms on the person-level led to a loss of information in comparison with the beep-level, as the variance of beeps is not reflected in the aggregated scores. Nonetheless, compared with a single questionnaire assessment, the aggregated experience sampling measures used in this study still provide higher levels of precision in measurement. The number of statistical analyses performed may have resulted in multiple testing problems. However, in order to control for type I error, results were corrected using the Simes method [52] by momentary manifestation of negative symptom and outcome domain. In addition, time to follow-up was used as a crude proxy to impute for time to remission from UHR status (eg, for participants who remitted at any time between baseline and 1-year follow-up, the date of the 1-year follow-up assessment was used as proxy), which might lead to imprecision in these exploratory survival analyses. Future research should attempt to establish a more precise data collection for time to remission.

### Comparison With Previous Research

To our knowledge, this is the first study using an experience sampling design to investigate the predictive value of momentary manifestations of negative symptoms measured in individuals UHR. In accordance with our hypotheses, we found evidence for more intense momentary manifestations of negative symptoms to be associated with poorer functioning and higher illness severity at follow-up. In addition, we found evidence that individuals with greater variability of positive affect (as a measure of blunted affective experience), experienced a shorter time to remission from UHR status. This is in line with findings from previous studies using other operationalizations of negative symptoms [2-5]. Given that ESM measures of momentary manifestations of negative symptoms are intended to capture subjective experience of social context, our findings primarily pertain to the experiential level rather than to the level of expression [27].

Our findings tentatively suggest that blunted affect, lack of social drive, and anhedonia are associated with some clinical outcomes, but findings on social anhedonia were most robust. We may speculate that changes in affective response to social contact (ie, social anhedonia) in daily life may be most relevant in individuals at UHR, whereas other types of momentary manifestations of negative symptoms (eg, lack of social drive) may be more relevant in later stages of psychosis. Social anhedonia may contribute to a loss of reinforcement of social contact, which might encourage a progressive decrease of social interaction and social functioning more downstream, closer to, or directly at, onset of psychotic disorder [35,57-59].

The findings have important implications for clinicians and researchers aiming to improve functional outcomes of individuals at UHR. Recent meta-analyses found no evidence for psychosocial treatment to improve functioning in individuals at UHR [60], with poor functioning at baseline being, in turn, a predictor for later psychopathology [61]. Taken together, this may contribute to a vicious cycle of symptom burden and poor functioning amplifying each other in this group at risk. Therefore, new intervention approaches are urgently required and the experience of momentary manifestations of negative symptoms, especially social anhedonia, in daily life may be a promising target. Possibly, improving social anhedonia may diminish social isolation, and thereby improve outcomes.

In addition, we found only weak correlations between momentary manifestations of negative symptoms and the BPRS scores, highlighting the relevance of participants’ subjective experience. These discrepancies may be interpreted in different ways. First, discrepancies may evolve due to varying modes of assessment and, hence, precision of measurement. Gerritsen, Bagby [37] claim that some negative symptoms may be associated with no or very limited subjective distress and, hence, difficult to measure via self-report. However, one may argue that aggregating multiple momentary measurements across several days may provide a more precise measure of affective and motivational processes than cross-sectional clinical interviews [27]. Second, the discrepancies may, in fact, reflect 2 distinct dimensions of negative symptoms (ie, experience vs expression), and therefore, relying on purely behavioral indicators in assessing negative symptoms may result in a more limited understanding of internal, experiential aspects [27]. Both interpretations highlight the potential of ESM as a diagnostic tool over and above traditional clinical measures of symptoms [62].

### Conclusions

We found evidence for momentary manifestations of negative symptoms, especially social anhedonia, to predict clinical outcomes at follow-up. These findings emphasize that the assessment of momentary manifestations of negative symptoms in individuals at UHR is of considerable potential value for both diagnostic assessment and early intervention. The assessment of momentary manifestations of negative symptoms may provide a more comprehensive picture of patients’ symptoms in the context of their daily life for clinicians and researchers and contribute to a better understanding of individuals’ subjective experience. In addition, the experience of momentary manifestations of negative symptoms, especially social anhedonia, in daily life may be a promising target for interventions aiming to improve clinical outcomes in the early stages of psychosis.

## Appendix

Multimedia Appendix 1 Convergent validity of momentary manifestations of negative symptoms and interviewer-rated measures of negative symptoms.

Multimedia Appendix 2 Data quality of clinical outcome measures.

Multimedia Appendix 3 Unadjusted analyses.

Multimedia Appendix 4 Sensitivity analysis with current depressive episode as an additional independent variable to control for potential confounding.

Multimedia Appendix 5 Sensitivity analysis with comorbid Axis-I disorder as an additional independent variable to control for potential confounding.

Multimedia Appendix 6 Comorbid Axis-I diagnoses at baseline.

Multimedia Appendix 7 Restricted analyses.

## Abbreviations

BPRS: Brief Psychiatric Rating Scale
ESM: Experience Sampling Methodology
GAF: Global Assessment of Functioning
UHR: ultrahigh risk

## References

[1] Azar M, Pruessner M, Baer LH, Iyer S, Malla AK, Lepage M (2018). A study on negative and depressive symptom prevalence in individuals at ultra-high risk for psychosis. Early Interv Psychiatry.

[2] Fulford D, Niendam TA, Floyd EG, Carter CS, Mathalon DH, Vinogradov S, Stuart BK, Loewy RL (2013). Symptom dimensions and functional impairment in early psychosis: more to the story than just negative symptoms. Schizophr Res.

[3] Schlosser DA, Campellone TR, Biagianti B, Delucchi KL, Gard DE, Fulford D, Stuart BK, Fisher M, Loewy RL, Vinogradov S (2015). Modeling the role of negative symptoms in determining social functioning in individuals at clinical high risk of psychosis. Schizophr Res.

[4] Svirskis T, Korkeila J, Heinimaa M, Huttunen J, Ilonen T, Ristkari T, Hietala J, Syvälahti E, McGlashan T, Vahlberg T, Salokangas RK (2007). Quality of life and functioning ability in subjects vulnerable to psychosis. Compr Psychiatry.

[5] Burton CZ, Tso IF, Carrión RE, Niendam T, Adelsheim S, Auther AM, Cornblatt BA, Carter CS, Melton R, Sale TG, Taylor SF, McFarlane WR (2019). Baseline psychopathology and relationship to longitudinal functional outcome in attenuated and early first episode psychosis. Schizophr Res.

[6] Glenthøj LB, Kristensen TD, Wenneberg C, Hjorthøj C, Nordentoft M (2020). Investigating cognitive and clinical predictors of real-life functioning, functional capacity, and quality of life in individuals at ultra-high risk for psychosis. Schizophr Bull Open.

[7] Demjaha A, Valmaggia L, Stahl D, Byrne M, McGuire P (2012). Disorganization/cognitive and negative symptom dimensions in the at-risk mental state predict subsequent transition to psychosis. Schizophr Bull.

[8] Piskulic D, Addington J, Cadenhead KS, Cannon TD, Cornblatt BA, Heinssen R, Perkins DO, Seidman LJ, Tsuang MT, Walker EF, Woods SW, McGlashan TH (2012). Negative symptoms in individuals at clinical high risk of psychosis. Psychiatry Res.

[9] Oliver D, Reilly T, Baccaredda Boy O, Petros N, Davies C, Borgwardt S, McGuire P, Fusar-Poli P (2020). What causes the onset of psychosis in individuals at clinical high risk? A meta-analysis of risk and protective factors. Schizophr Bull.

[10] Valmaggia LR, Stahl D, Yung AR, Nelson B, Fusar-Poli P, McGorry PD, McGuire PK (2013). Negative psychotic symptoms and impaired role functioning predict transition outcomes in the at-risk mental state: a latent class cluster analysis study. Psychol Med.

[11] Zimmermann R, Gschwandtner U, Wilhelm FH, Pflueger MO, Riecher-Rössler A, Fuhr P (2010). EEG spectral power and negative symptoms in at-risk individuals predict transition to psychosis. Schizophr Res.

[12] Alderman T, Addington J, Bearden C, Cannon TD, Cornblatt BA, McGlashan TH, Perkins DO, Seidman LJ, Tsuang MT, Walker EF, Woods SW, Cadenhead KS (2015). Negative symptoms and impaired social functioning predict later psychosis in Latino youth at clinical high risk in the North American prodromal longitudinal studies consortium. Early Interv Psychiatry.

[13] Ferrarelli F, Mathalon D (2020). The prodromal phase: time to broaden the scope beyond transition to psychosis?. Schizophr Res.

[14] Simon AE, Velthorst E, Nieman DH, Linszen D, Umbricht D, de Haan L (2011). Ultra high-risk state for psychosis and non-transition: a systematic review. Schizophr Res.

[15] Fusar-Poli P, Bonoldi I, Yung AR, Borgwardt S, Kempton MJ, Valmaggia L, Barale F, Caverzasi E, McGuire P (2012). Predicting psychosis: meta-analysis of transition outcomes in individuals at high clinical risk. Arch Gen Psychiatry.

[16] Addington J, Liu L, Buchy L, Cadenhead K, Cannon T, Cornblatt B, Perkins DO, Seidman LJ, Tsuang MT, Walker EF, Woods SW, Bearden CE, Mathalon DH, McGlashan TH (2015). North American Prodrome Longitudinal Study (NAPLS 2): the prodromal symptoms. J Nerv Ment Dis.

[17] Polari A, Lavoie S, Yuen H, Amminger P, Berger G, Chen E, deHaan L, Hartmann J, Markulev C, Melville F, Nieman D, Nordentoft M, Riecher-Rössler A, Smesny S, Stratford J, Verma S, Yung A, McGorry P, Nelson B (2018). Clinical trajectories in the ultra-high risk for psychosis population. Schizophr Res.

[18] Addington J, Cornblatt BA, Cadenhead KS, Cannon TD, McGlashan TH, Perkins DO, Seidman LJ, Tsuang MT, Walker EF, Woods SW, Heinssen R (2011). At clinical high risk for psychosis: outcome for nonconverters. Am J Psychiatry.

[19] Simon AE, Borgwardt S, Riecher-Rössler A, Velthorst E, de Haan L, Fusar-Poli P (2013). Moving beyond transition outcomes: meta-analysis of remission rates in individuals at high clinical risk for psychosis. Psychiatry Res.

[20] Fusar-Poli P, Rocchetti M, Sardella A, Avila A, Brandizzi M, Caverzasi E, Politi P, Ruhrmann S, McGuire P (2015). Disorder, not just state of risk: meta-analysis of functioning and quality of life in people at high risk of psychosis. Br J Psychiatry.

[21] Lin A, Wood SJ, Nelson B, Beavan A, McGorry P, Yung AR (2015). Outcomes of nontransitioned cases in a sample at ultra-high risk for psychosis. Am J Psychiatry.

[22] Beck K, Andreou C, Studerus E, Heitz U, Ittig S, Leanza L, Riecher-Rössler A (2019). Clinical and functional long-term outcome of patients at clinical high risk (CHR) for psychosis without transition to psychosis: a systematic review. Schizophr Res.

[23] Michel C, Ruhrmann S, Schimmelmann BG, Klosterkötter J, Schultze-Lutter F (2018). Course of clinical high-risk states for psychosis beyond conversion. Eur Arch Psychiatry Clin Neurosci.

[24] Kay SR, Fiszbein A, Opler LA (1987). The positive and negative syndrome scale (PANSS) for schizophrenia. Schizophr Bull.

[25] Kay SR, Opler LA, Lindenmayer J (1989). The Positive and Negative Syndrome Scale (PANSS): rationale and standardisation. Br J Psychiatry Suppl.

[26] Oorschot M, Lataster T, Thewissen V, Lardinois M, Wichers M, van Os J, Delespaul P, Myin-Germeys I (2013). Emotional experience in negative symptoms of schizophrenia--no evidence for a generalized hedonic deficit. Schizophr Bull.

[27] Blanchard JJ, Shan L, Andrea A, Savage C, Kring AM, Weittenhiller L, Badcock JC, Paulik-White G (2020). Negative symptoms and their assessment in schizophrenia and related disorders. A Clinical Introduction to Psychosis.

[28] Galderisi S, Mucci A, Buchanan RW, Arango C (2018). Negative symptoms of schizophrenia: new developments and unanswered research questions. Lancet Psychiatry.

[29] Kring AM, Caponigro JM (2010). Emotion in schizophrenia: where feeling meets thinking. Curr Dir Psychol Sci.

[30] Cohen AS, Najolia GM, Brown LA, Minor KS (2011). The state-trait disjunction of anhedonia in schizophrenia: potential affective, cognitive and social-based mechanisms. Clin Psychol Rev.

[31] Myin-Germeys I, Kasanova Z, Vaessen T, Vachon H, Kirtley O, Viechtbauer W, Reininghaus U (2018). Experience sampling methodology in mental health research: new insights and technical developments. World Psychiatry.

[32] Mote J, Fulford D (2020). Ecological momentary assessment of everyday social experiences of people with schizophrenia: a systematic review. Schizophr Res.

[33] Myin-Germeys I, Delespaul PA, deVries MW (2000). Schizophrenia patients are more emotionally active than is assumed based on their behavior. Schizophr Bull.

[34] Dejonckheere E, Mestdagh M, Houben M, Rutten I, Sels L, Kuppens P, Tuerlinckx F (2019). Complex affect dynamics add limited information to the prediction of psychological well-being. Nat Hum Behav.

[35] Hermans KS, Myin-Germeys I, Gayer-Anderson C, Kempton MJ, Valmaggia L, McGuire P, Murray RM, Garety P, Wykes T, Morgan C, Kasanova Z, Reininghaus U (2020). Elucidating negative symptoms in the daily life of individuals in the early stages of psychosis. Psychol Med.

[36] Kwapil TR, Silvia PJ, Myin-Germeys I, Anderson A, Coates SA, Brown LH (2009). The social world of the socially anhedonic: exploring the daily ecology of asociality. J Res Pers.

[37] Gerritsen C, Bagby RM, Sanches M, Kiang M, Maheandiran M, Prce I, Mizrahi R (2019). Stress precedes negative symptom exacerbations in clinical high risk and early psychosis: a time-lagged experience sampling study. Schizophr Res.

[38] Aghevli MA, Blanchard JJ, Horan WP (2003). The expression and experience of emotion in schizophrenia: a study of social interactions. Psychiatry Res.

[39] van Os J, Rutten BP, Myin-Germeys I, Delespaul P, Viechtbauer W, van Zelst C, Bruggeman R, Reininghaus U, Morgan C, Murray RM, Di Forti M, McGuire P, Valmaggia LR, Kempton MJ, Gayer-Anderson C, Hubbard K, Beards S, Stilo SA, Onyejiaka A, Bourque F, Modinos G, Tognin S, Calem M, O'Donovan MC, Owen MJ, Holmans P, Williams N, Craddock N, Richards A, Humphreys I, Meyer-Lindenberg A, Leweke FM, Tost H, Akdeniz C, Rohleder C, Bumb JM, Schwarz E, Alptekin K, Üçok A, Saka MC, Atbaşoğlu EC, Gülöksüz S, Gumus-Akay G, Cihan B, Karadağ H, Soygür H, Cankurtaran ES, Ulusoy S, Akdede B, Binbay T, Ayer A, Noyan H, Karadayı G, Akturan E, Ulaş H, Arango C, Parellada M, Bernardo M, Sanjuán J, Bobes J, Arrojo M, Santos JL, Cuadrado P, Rodríguez Solano JJ, Carracedo A, García Bernardo E, Roldán L, López G, Cabrera B, Cruz S, Díaz Mesa EM, Pouso M, Jiménez E, Sánchez T, Rapado M, González E, Martínez C, Sánchez E, Olmeda MS, de Haan L, Velthorst E, van der Gaag M, Selten J, van Dam D, van der Ven E, van der Meer F, Messchaert E, Kraan T, Burger N, Leboyer M, Szoke A, Schürhoff F, Llorca P, Jamain S, Tortelli A, Frijda F, Vilain J, Galliot A, Baudin G, Ferchiou A, Richard J, Bulzacka E, Charpeaud T, Tronche A, De Hert M, van Winkel R, Decoster J, Derom C, Thiery E, Stefanis NC, Sachs G, Aschauer H, Lasser I, Winklbaur B, Schlögelhofer M, Riecher-Rössler A, Borgwardt S, Walter A, Harrisberger F, Smieskova R, Rapp C, Ittig S, Soguel-dit-Piquard F, Studerus E, Klosterkötter J, Ruhrmann S, Paruch J, Julkowski D, Hilboll D, Sham PC, Cherny SS, Chen EY, Campbell DD, Li M, Romeo-Casabona CM, Emaldi Cirión A, Urruela Mora A, Jones P, Kirkbride J, Cannon M, Rujescu D, Tarricone I, Berardi D, Bonora E, Seri M, Marcacci T, Chiri L, Chierzi F, Storbini V, Braca M, Minenna MG, Donegani I, Fioritti A, La Barbera D, La Cascia CE, Mulè A, Sideli L, Sartorio R, Ferraro L, Tripoli G, Seminerio F, Marinaro AM, McGorry P, Nelson B, Amminger GP, Pantelis C, Menezes PR, Del-Ben CM, Gallo Tenan SH, Shuhama R, Ruggeri M, Tosato S, Lasalvia A, Bonetto C, Ira E, Nordentoft M, Krebs M, Barrantes-Vidal N, Cristóbal P, Kwapil TR, Brietzke E, Bressan RA, Gadelha A, Maric NP, Andric S, Mihaljevic M, Mirjanic T, European Network of National Networks studying Gene-Environment Interactions in Schizophrenia (EU-GEI) (2014). Identifying gene-environment interactions in schizophrenia: contemporary challenges for integrated, large-scale investigations. Schizophr Bull.

[40] Yung AR, Yuen HP, McGorry PD, Phillips LJ, Kelly D, Dell'Olio M, Francey SM, Cosgrave EM, Killackey E, Stanford C, Godfrey K, Buckby J (2005). Mapping the onset of psychosis: the Comprehensive Assessment of At-Risk Mental States. Aust N Z J Psychiatry.

[41] Myin-Germeys I, Oorschot M, Collip D, Lataster J, Delespaul P, van Os J (2009). Experience sampling research in psychopathology: opening the black box of daily life. Psychol Med.

[42] Palmier-Claus JE, Myin-Germeys I, Barkus E, Bentley L, Udachina A, Delespaul PA, Lewis SW, Dunn G (2011). Experience sampling research in individuals with mental illness: reflections and guidance. Acta Psychiatr Scand.

[43] Shiffman S, Stone AA, Hufford MR (2008). Ecological momentary assessment. Annu Rev Clin Psychol.

[44] Palmier-Claus JE, Dunn G, Lewis SW (2012). Emotional and symptomatic reactivity to stress in individuals at ultra-high risk of developing psychosis. Psychol Med.

[45] Overall JE, Gorham DR (1962). The brief psychiatric rating scale. Psychol Rep.

[46] (1994). Diagnostic and Statistical Manual of Mental Disorders: DSM-IV [Internet]. 4th edition.

[47] Guy W (1976). ECDEU assessment manual for psychopharmacology. US Dept Health, Education, and Welfare, Public Health Service, Alcohol, Drug Abuse, and Mental Health Administration, National Institute of Mental Health, Psychopharmacology Research Branch, Division of Extramural Research Programs.

[48] McManus MD, Siegel JT, Nakamura J (2019). The predictive power of low-arousal positive affect. Motiv Emot.

[49] Delespaul P, deVries M, van Os J (2002). Determinants of occurrence and recovery from hallucinations in daily life. Soc Psychiatry Psychiatr Epidemiol.

[50] Janssens M, Lataster T, Simons C, Oorschot M, Lardinois M, van Os J, Myin-Germeys I, GROUP (2012). Emotion recognition in psychosis: no evidence for an association with real world social functioning. Schizophr Res.

[51] van Winkel R, Henquet C, Rosa A, Papiol S, Fananás L, De Hert M, Peuskens J, van Os J, Myin-Germeys I (2008). Evidence that the COMT(Val158Met) polymorphism moderates sensitivity to stress in psychosis: an experience-sampling study. Am J Med Genet B Neuropsychiatr Genet.

[52] Simes RJ (1986). An improved Bonferroni procedure for multiple tests of significance. Biometrika.

[53] Lim J, Rekhi G, Rapisarda A, Lam M, Kraus M, Keefe RS, Lee J (2015). Impact of psychiatric comorbidity in individuals at Ultra High Risk of psychosis - findings from the Longitudinal Youth at Risk Study (LYRIKS). Schizophr Res.

[54] Albert U, Tomassi S, Maina G, Tosato S (2018). Prevalence of non-psychotic disorders in ultra-high risk individuals and transition to psychosis: a systematic review. Psychiatry Res.

[55] Malda A, Boonstra N, Barf H, de Jong S, Aleman A, Addington J, Pruessner M, Nieman D, de Haan L, Morrison A, Riecher-Rössler A, Studerus E, Ruhrmann S, Schultze-Lutter F, An SK, Koike S, Kasai K, Nelson B, McGorry P, Wood S, Lin A, Yung AY, Kotlicka-Antczak M, Armando M, Vicari S, Katsura M, Matsumoto K, Durston S, Ziermans T, Wunderink L, Ising H, van der Gaag M, Fusar-Poli P, Pijnenborg GH (2019). Individualized prediction of transition to psychosis in 1,676 individuals at clinical high risk: development and validation of a multivariable prediction model based on individual patient data meta-analysis. Front Psychiatry.

[56] Nelson B, Yuen HP, Wood SJ, Lin A, Spiliotacopoulos D, Bruxner A, Broussard C, Simmons M, Foley DL, Brewer WJ, Francey SM, Amminger GP, Thompson A, McGorry PD, Yung AR (2013). Long-term follow-up of a group at ultra high risk ("prodromal") for psychosis: the PACE 400 study. JAMA Psychiatry.

[57] Corcoran CM, Kimhy D, Parrilla-Escobar MA, Cressman VL, Stanford AD, Thompson J, David SB, Crumbley A, Schobel S, Moore H, Malaspina D (2011). The relationship of social function to depressive and negative symptoms in individuals at clinical high risk for psychosis. Psychol Med.

[58] Reininghaus U, Kempton MJ, Valmaggia L, Craig TK, Garety P, Onyejiaka A, Gayer-Anderson C, So SH, Hubbard K, Beards S, Dazzan P, Pariante C, Mondelli V, Fisher HL, Mills JG, Viechtbauer W, McGuire P, van Os J, Murray RM, Wykes T, Myin-Germeys I, Morgan C (2016). Stress sensitivity, aberrant salience, and threat anticipation in early psychosis: an experience sampling study. Schizophr Bull.

[59] Edwards CJ, Cella M, Emsley R, Tarrier N, Wykes TH (2018). Exploring the relationship between the anticipation and experience of pleasure in people with schizophrenia: an experience sampling study. Schizophr Res.

[60] Devoe DJ, Farris MS, Townes P, Addington J (2019). Interventions and social functioning in youth at risk of psychosis: a systematic review and meta-analysis. Early Interv Psychiatry.

[61] Velthorst E, Nelson B, Wiltink S, de Haan L, Wood SJ, Lin A, Yung AR (2013). Transition to first episode psychosis in ultra high risk populations: does baseline functioning hold the key?. Schizophr Res.

[62] van Os J, Lataster T, Delespaul P, Wichers M, Myin-Germeys I (2014). Evidence that a psychopathology interactome has diagnostic value, predicting clinical needs: an experience sampling study. PLoS One.

