# Supplementary material for: Momentary Manifestations of Negative Symptoms as Predictors of Clinical Outcomes in People at High Risk for Psychosis: Experience Sampling Study
Source: JMIR Ment Health. 2021 Nov 19;8(11):e30309. doi: 10.2196/30309 (PMC8663470; doi:10.2196/30309)
Supplement: Multimedia Appendix 1 [file mental_v8i11e30309_app1.docx]

# Supplementary Material 1

## Convergent validity of momentary manifestations of negative symptoms and interviewer-rated measures of negative symptoms

The association of momentary manifestations of negative symptoms and observer-rated measures of negative symptoms at baseline was examined to evaluate convergent validity of the measures. In addition, we used observer-rated measures of negative symptoms to predict momentary manifestations of negative symptoms measured with ESM in a multilevel model.

**Table S1.** Correlation matrix of momentary manifestations of negative symptoms and observer-rated negative symptoms.

|  | BPRS**^a^** total score | BPRS neg. symptom score | Intensity NA**^b^** | Intensity PA**^c^** | Variability NA | Variability PA | Instability  NA | Instability  PA | Anhedonia | Social Anhedonia | Amount of time spent alone | Preference to be alone when in company | Pleasantness of being alone |
| --- | --- | --- | --- | --- | --- | --- | --- | --- | --- | --- | --- | --- | --- |
|  |  |  |  |  |  |  |  |  |  |  |  |  |  |
| BPRS total score | 1.00 |  |  |  |  |  |  |  |  |  |  |  |  |
| BPRS neg. symptom score | 0.54  *p*<.001 | 1.00 |  |  |  |  |  |  |  |  |  |  |  |
| Intensity NA | 0.28  *p*=.017 | 0.13  *p*=.285 | 1.00 |  |  |  |  |  |  |  |  |  |  |
| Intensity  PA | -0.34  *p*=.004 | -0.21  *p*=.081 | -0.60  *p*<.001 | 1.00 |  |  |  |  |  |  |  |  |  |
| Variability NA | 0.26  *p*=.025 | 0.03  *p*=.777 | 0.28  *p*=.012 | -0.06  *p*=.590 | 1.00 |  |  |  |  |  |  |  |  |
| Variability PA | -0.06  *p*=.595 | -0.10  *p*=.418 | 0.03  *p*=.823 | 0.18  *p*=.112 | 0.67  *p*<.001 | 1.00 |  |  |  |  |  |  |  |
| Instability  NA | 0.18  *p*=.123 | 0.09  *p*=.420 | 0.09  *p*=.420 | 0.06  *p*=.621 | 0.85  *p*<.001 | 0.62  *p*<.001 | 1.00 |  |  |  |  |  |  |
| Instability  PA | 0.00  *p*=.982 | -0.13  *p*=.270 | -0.08  *p*=.497 | .23  *p*=.038 | 0.61  *p*<.001 | 0.80  *p*<.001 | 0.78  *p*<.001 | 1.00 |  |  |  |  |  |
| Anhedonia | -0.34  *p*=.003 | -0.19  *p*=.100 | -0.57  *p*<.001 | 0.99  *p*<.001 | -0.01  *p*=.908 | 0.25  *p*=.028 | 0.09  *p*=.427 | 0.28  *p*=.012 | 1.00 |  |  |  |  |
| Social Anhedonia | -0.31  *p*=.008 | -0.17  *p*=.142 | -0.53  *p*<.001 | 0.95  *p*<.001 | -0.07  *p*=.541 | 0.20  *p*=.07 | 0.03  *p*=.781 | 0.21  *p*=.060 | 0.95  *p*<.001 | 1.00 |  |  |  |
| Amount of time spent alone | -0.13  *p*=.266 | -0.12  *p*=.327 | -0.05  *p*=.687 | 0.08  *p*=.458 | 0.01  *p*=.944 | 0.12  *p*=.283 | 0.11  *p*=.320 | 0.16  *p*=.155 | 0.11  *p*=.314 | 0.12  *p*=.309 | 1.00 |  |  |
| Preference to be alone when in company | 0.10  *p*=.412 | 0.16  *p*=.183 | .049  *p*<.001 | -0.46  *p*<.001 | 0.06  *p*=.576 | -0.12  *p*=.294 | -0.02  *p*=.880 | -0.11  *p*=.342 | -0.470  *p*<.001 | -0.50  *p*<.001 | -0.20  *p*=.074 | 1.00 |  |
| Pleasantness of being alone | -0.08  *p*=.489 | -0.03  *p*=.789 | 0.02  *p*=.894 | 0.06  *p*=.613 | 0.15  *p*=.184 | 0.00  *p*=.999 | 0.14  *p*=.232 | 0.04  *p*=.714 | 0.04  *p*=.752 | -0.03  *p*=.786 | -0.05  *p*=.661 | 0.42  *p*=.002 | 1.00 |

^a^ Brief Psychiatric Rating Scale.

^b^ NA, negative affect

^c^ PA, positive affect.

Table S2. Momentary manifestations of negative symptoms predicted by observer-rated negative symptoms.

|  | **Predictors** | | | |
| --- | --- | --- | --- | --- |
|  | **BPRS** ^a^ **total score** | | **BPRS negative symptom score** | |
|  | *b* (CI ^b^) | *p* | *b* (CI) | *p* |
|  |  |  |  |  |
| Outcome: Blunted affective experience | | | | |
| Intensity NA ^c^ | 0.04  (0.01 – 0.06) | .013 | 0.08  (-0.07 – 0.23) | .292 |
| Intensity PA ^d^ | -0.04  (-0.06 - -0.02) | <.001 | -0.11  (-0.23 – 0.01) | .063 |
| Instability NA | 0.04  (0.00 – 0.08) | .029 | 0.01  (-0.18 – 0.20) | .905 |
| Instability PA | 0.00  (-0.02 – 0.03) | .965 | -0.06  (-0.19 – 0.06) | .332 |
| Variability NA | 0.03  (0.01 – 0.04) | .003 | 0.02  (-0.08 – 0.11) | .731 |
| Variability PA | 0.00  (-0.02 – 0.01.) | .639 | -0.03  (-0.12 – 0.05) | .471 |
| Outcome: Lack of social drive | | | | |
| Preference to be alone when   in company | 0.01  (-0.02 – 0.04) | .450 | 0.12  (-0.05 – 0.28) | .165 |
| Pleasantness of being alone | -0.01  (-0.05 – 0.02) | .480 | -0.02  (-0.20 – 0.16) | .800 |
| Outcome: Anhedonia | -0.04  (-0.06 – -0.02) | <.001 | -0.09  (-0.20 – 0.02) | .095 |
| Outcome: Social Anhedonia | -0.04  (-0.06 – -0.01) | .001 | -0.08  (-0.19 – 0.03) | .134 |

^a^ Brief Psychiatric Rating Scale.

^b^ CI, confidence interval.

^c^ NA, negative affect.

^d^ PA, positive affect.
