# Supplementary material for: Momentary Manifestations of Negative Symptoms as Predictors of Clinical Outcomes in People at High Risk for Psychosis: Experience Sampling Study
Source: JMIR Ment Health. 2021 Nov 19;8(11):e30309. doi: 10.2196/30309 (PMC8663470; doi:10.2196/30309)
Supplement: Multimedia Appendix 2 [file mental_v8i11e30309_app2.docx]

# Supplementary Material 2

## Data quality of clinical outcome measures

To ensure data quality, extensive training on instruments and interview skills was provided. Initial assessments were reviewed, and possible difficulties were anticipated. In addition to the EU-GEI web-based training designed to control and increase inter-rater reliability, regular meetings were held to discuss case vignettes. Site visits were held in order to evaluate and standardize interviews. In addition, extensive, repetitious training procedures and reliability checks were conducted. Training videos of the most advanced instruments were updated regularly. For each of the training videos, a ‘gold standard score’ was determined through independent rating of the training videos by independent experienced researchers. In case of disagreement, the head of the training work package was consulted. Per instrument, we subsequently determined the maximum number of errors/ deviation from the gold standard score the researcher was allowed, in order to ‘pass’ the video.
