# Supplementary material for: Momentary Manifestations of Negative Symptoms as Predictors of Clinical Outcomes in People at High Risk for Psychosis: Experience Sampling Study
Source: JMIR Ment Health. 2021 Nov 19;8(11):e30309. doi: 10.2196/30309 (PMC8663470; doi:10.2196/30309)
Supplement: Multimedia Appendix 3 [file mental_v8i11e30309_app3.docx]

# Supplementary Material 3

## Unadjusted analyses

Table S3. Clinical outcomes at 1- and 2-year follow-up predicted by blunted affective experience at baseline (i.e., intensity, instability and variability of negative and positive affect) and clinical outcome at baseline – unadjusted analyses.

|  | | | | | Level of functioning: Symptoms ^a^ | | | | | | Level of functioning: Disability | | | | | | |
| --- | --- | --- | --- | --- | --- | --- | --- | --- | --- | --- | --- | --- | --- | --- | --- | --- | --- |
|  | | | | | 1-year follow-up *(N=48)* | | | | 2-year follow-up *(N=36)* | | 1-year follow-up *(N=48)* | | | | | 2-year follow-up *(N=36)* | |
|  | | | | | *b* (CI ^b^) | | *p* | | *b* (CI) | *p* | *b* (CI) | | *p* | | *b* (CI) | | *p* |
|  | | | | |  | |  | |  |  |  | |  | |  | |  |
| Predictor: Intensity NA ^c^ | | | | | | | | | | | | | | | | | |
| Outcome at baseline | | | | | 0.25  (-0.03 – 0.54) | | .077 | | 0.26  (-0.23 – 0.75) | .285 | 0.42 (0.15 – 0.70) | | .003 | | 0.49 (0.11 – 0.87) | | .013 |
| Intensity NA | | | | | -3.31  (-6.47 – -1.41) | | .041 | | -2.94  (-6.69 – 0.80) | .119 | -3.81 (-7.85 – 0.04) | | .048 | | 0.80 (-3.44 – 5.04) | | .701 |
| Predictor: Intensity PA ^d^ | | | | | | | | | | | | | | | | | |
| Outcome at baseline | | | | | 0.26  (-0.03 – 0.55) | | .075 | | 0.29  (-0.20 – 0.79) | .240 | 0.39  (0.12 – 0.66) | | .006 | | 0.50  (0.12 – 0.87) | | .011 |
| Intensity PA | | | | | 3.03  (-0.62 – 6.67) | | .101 | | 2.08  (-2.46 – 6.63) | .385 | 5.48  (1.34 – 9.62) | | .011 | | 1.46  (-3.64 – 6.55) | | .562 |
| Predictor: Instability NA | | | | | | | | | | | | | | | | | |
| Outcome at baseline | | | | | 0.30  (0.00 – 0.60) | | .052 | | 0.22  (-0.28 – 0.72) | .374 | 0.40  (0.11 – 0.68) | | .008 | | 0.38  (0.01 – 0.75) | | .045 |
| Instability NA | | | | | -0.02  (-1.84 – 1.81) | | .986 | | -2.55  (-5.95 – 0.85) | .136 | -0.87  (-3.01 – 1.27) | | .417 | | -3.01  (-6.70 – 0.69) | | .107 |
| Predictor: Instability PA | | | | | | | | | | | | | | | | | |
| Outcome at baseline | | | | | 0.28  (-0.01 – 0.58) | | .061 | | 0.17  (-0.31 – 0.66) | .471 | 0.41  (0.12 – 0.70) | | .006 | | 0.38  (0.02 – 0.74) | | .039 |
| Instability PA | | | | | -1.24  (-4.19 – 1.70) | | .400 | | -5.40  (-10.65 – -0.16) | .044 | -0.95  (-4.46 – 2.56) | | .588 | | -6.40  (-12.01 – -0.79) | | .027 |
| Predictor: Variability NA | | | | | | | | | | | | | | | | | |
| Outcome at baseline | | | | | 0.29  (-0.01 – 0.58) | | .059 | | 0.21  (-0.27 – 0.69) | .376 | 0.40  (0.11 – 0.69) | | .007 | | 0.41  (0.04 – 0.77) | | .032 |
| Variability NA | | | | | -0.92  (-4.70 – 2.78) | | .628 | | -5.50  (-10.96 – -0.05) | .048 | -1.97  (-6.41 – 2.48) | | .378 | | -5.67  (-11.74 – 0.40) | | .066 |
| Predictor: Variability PA | | | | | | | | | | | | | | | | | |
| Outcome at baseline | | | | | 0.29  (0.00 – 0.58) | | .049 | | 0.26  (-0.21 - 0.74) | .266 | 0.41  (0.12 – 0.70) | | .007 | | 0.37  (0.00 – 0.75) | | .051 |
| Variability PA | | | | | -1.68  (-6.01– 2.65) | | .438 | | -5.86  (-11.79 – 0.08) | .053 | -0.62  (-5.83 – 4.60) | | .813 | | -5.13  (-11.91 – 1.65) | | .134 |
|  | | **Illness severity** ^e^ | | | | | | | | | | **Remission from UHR status** | | | | **Transition status** | |
|  | | 1-year follow-up *(N=47)* | | | | | | 2-year follow-up *(N=37)* | | | | *(N=54)* | | | | *(N=57)* | |
|  |  | *b* (CI) | | | | *p* | | *b* (CI) | | *p* | | *HR* ^f^ (CI) | | *p* | | *HR* (CI) | *p* |
| Predictor: Intensity NA | | | | | | | | | | | | | | | | | |
| Outcome at  baseline | | 0.62 (0.37 – 0.88) | | | | <.001 | | 0.46 (0.11 – 0.80) | | .011 | |  | |  | |  | |
| Intensity NA | | 0.36 (0.02 – 0.69) | | | | .036 | | 0.00 (-0.38 – 0.37) | | .992 | | 0.33  (0.17 – 0.63) | | .001 | | 1.73  (0.94 – 3.21) | .080 |
| Predictor: Intensity PA | | | | | | | | | | | | | | | | | |
| Outcome at  baseline | | 0.62  (0.35 – 0.89) | | | | .<001 | | 0.36  (0.02 – 0.71) | | .039 | |  | |  | |  |  |
| Intensity PA | | -0.31  (-0.68 – 0.06) | | | | .103 | | -0.40  (-0.85 – 0.06) | | .083 | | 2.40  (1.16 – 4.98) | | . 018 | | 0.61  (0.27 – 1.40) | .245 |
| Predictor: Instability NA | | | | | | | | | | | | | | | | | |
| Outcome at  baseline | | 0.68  (0.42 – 0.94) | | | | <.001 | | 0.46  (0.12 – 0.81) | | .010 | |  | |  | |  | |
| Instability NA | | -0.04  (-0.22 – 0.14) | | | | .648 | | 0.03  (-0.31 – 0.37) | | .853 | | 0.87  (0.44 – 1.72) | | ..678 | | 1.10  (0.78 – 1.55) | .594 |
| Predictor: Instability PA | | | | | | | | | | | | | | | | | |
| Outcome at  baseline | | | 0.68  (0.42 – 0.95) | | | <.001 | | 0.48  (0.14 – 0.82) | | .007 | |  | |  | |  | |
| Instability PA | | | -0.04  (-0.33 – 0.26) | | | .797 | | 0.23  (-0.28 – 0.74) | | .366 | | 1.37  (0.70 – 2.68) | | .353 | | 1.25  (0.69 – 2.24) | .465 |
| Predictor: Variability NA | | | | | | | | | | | | | | | | | |
| Outcome at  baseline | | | | 0.68  (0.42 – 0.95) | | <.001 | | 0.47  (0.11 – 0.82) | | .011 | |  | |  | |  | |
| Variability NA | | | | -0.03  (-0.40 – 0.35) | | .882 | | 0.06  (-0.53 – 0.64) | | .840 | | 0.62  (0.16 – 2.35) | | .485 | | 1.43  (0.77 – 2.64) | .258 |
| Predictor: Variability PA | | | | | | | | | | | | | | | | | |
| Outcome at baseline | | | | 0.72  (0.45 – 0.98) | | <.001 | | 0.55  (0.20 – 0.89) | | .003 | |  | |  | |  | |
| Variability PA | | | | 0.20  (-0.24 – 0.64) | | .371 | | 0.52  (-0.10 – 1.15) | | .098 | | 2.10  (0.98 – 4.50) | | .058 | | 1.84  (0.83 – 4.08) | .136 |

^a^ Level of functioning assessed with the Global Assessment of Functioning Scale.

^b^ CI, confidence interval.

^c^ NA, negative affect.

^d^ PA, positive affect.

^e^ Illness severity assessed with the Clinical Global Impression Scale.

^f^ HR, Hazard ratio.

Table S4. Clinical outcomes at 1- and 2-year follow-up predicted by lack of social drive (i.e., amount of time spent alone, preference to be alone when in company and experienced pleasantness of being alone) and clinical outcome at baseline – unadjusted analyses.

|  | | Level of functioning: Symptoms ^a^ | | | | | | Level of functioning: Disability | | | | | | | | | | | | |  |
| --- | --- | --- | --- | --- | --- | --- | --- | --- | --- | --- | --- | --- | --- | --- | --- | --- | --- | --- | --- | --- | --- |
|  | | 1-year follow-up *(N=48)* | | | 2-year follow-up *(N=36)* | | | 1-year follow-up *(N=48)* | | | | | | | 2-year follow-up *(N=36)* | | | | | |  |
|  | | *b* (CI ^b^) | *p* | | *b* (CI) | *p* | | *b* (CI) | | | | | *p* | | *b* (CI) | | | | *p* | |  |
|  | |  |  | |  |  | |  | | | | |  | |  | | | |  | |  |
| Predictor: Amount of time spent alone | | | | | | | | | | | | | | | | | | | | | |
| Outcome at baseline | | 0.30  (0.00 – 0.59) | | .046 | 0.26  (-0.23 – 0.75) | .282 | | 0.39  (0.13 – 0.65) | | | | | .004 | | 0.31  (-0.06 – 0.68) | | | | .097 | |  |
| Amount of time spent alone | | 2.51  (-9.29 – 14.31) | | .670 | 12.06  (-4.51 – 28.63) | .148 | | 4.32  (-9.03 – 17.67) | | | | | .519 | | 19.01  (0.87 – 37.15) | | | | .041 | |  |
| Predictor: Preference to be alone when in company | | | | | | | | | | | | | | | | | | | | | |
| Outcome at baseline | | 0.27  (-0.02 – 0.56) | .064 | | 0.34  (-0.15 – 0.83) | .168 | | 0.40  (0.12 – 0.68) | | | | | .006 | | 0.38  (-0.01 – 0.76) | | | | .055 | |  |
| Preference to be alone | | -1.95  (-4.37 – 0.47) | .112 | | -2.66  (-6.29 – 0.97) | .146 | | -2.33  (-5.22 – 0.56) | | | | | .111 | | -1.04  (-5.20 – 3.12) | | | | .616 | |  |
| Predictor: Pleasantness of being alone | | | | | | | | | | | | | | | | | | | | | |
| Outcome at baseline | | 0.29  (-0.02 – 0.60) | .062 | | 0.32  (-0.18 – 0.81) | .203 | | 0.44  (0.15 – 0.73) | | | | | .004 | | 0.35  (-0.03 – 0.73) | | | | .067 | |  |
| Pleasantness of being alone | | -0.49  (-2.92 – 1.94) | .688 | | -1.68  (-4.82 – 1.46) | .284 | | -1.78  (-4.60 – 1.04) | | | | | .209 | | -2.54  (-6.02– 0.94) | | | | .147 | |  |
|  | | **Illness severity** ^c^ | | | | | | | | **Remission from**  **UHR status** | | | | | | | **Transition status** | | | | |
|  | | 1-year follow-up *(N=47)* | | | 2-year follow-up *(N=37)* | | | | | *(N=54)* | | | | | | | *(N=57)* | | | | |
|  |  | *b* (CI) | *p* | | *b* (CI) | | *p* | | *HR* ^d^ (CI) | | | | | *p* | *HR* (CI) | | | | | *p* | |
| Predictor: Amount of time spent alone | | | | | | | | | | | | | | | | | | | | | |
| Outcome at  baseline | | 0.70  (0.43 – 0.96) | <.001 | | 0.45  (0.11 – 0.79) | .011 | | |  | |  | | |  | |  | | | |  | |
| Amount of time spent alone | | 0.33  (-0.85 – 1.52) | .573 | | -0.60  (-2.23 – 1.04) | .464 | | | 1.77  (0.14 – 21.90) | | | | | .658 | | 0.10  (0.01 – 1.28) | | | | .076 | |
| Predictor: Preference to be alone when in company | | | | | | | | | | | | | | | | | | | | | |
| Outcome at  baseline | | 0.65  (0.38 – 0.92) | <.001 | | 0.45  (0.11 – 0.78) | .011 | | |  | | |  | |  | |  | |  | |  | |
| Preference to  be alone | | 0.14  (-0.11 – 0.40) | .267 | | 0.16  (-0.19 – 0.51) | .366 | | | 0.89  (0.51 – 1.53) | | | | | .665 | | 1.26  (0.72 – 2.21) | | | | .423 | |
| Predictor: Pleasantness of being alone | | | | | | | | | | | | | | | | | | | | | |
| Outcome at  baseline | | 0.67  (0.40 – 0.94) | <.001 | | 0.46  (0.13 – 0.80) | .009 | | |  | | |  | |  | |  | |  | | | |
| Pleasantness of being alone | | 0.14  (-0.10 – 0.38) | .234 | | 0.13  (-0.16 – 0.43) | .361 | | | 1.89  (0.55 – 1.73) | | | | | .940 | | 1.06  (0.61 – 1.84) | | | | .840 | |

^a^ Level of functioning assessed with the Global Assessment of Functioning Scale

^b^ CI, confidence interval.

^c^ Symptom severity assessed with the Clinical Global Impression Scale.

^d^ HR, Hazard ratio.

Table S5. Clinical Outcomes at 1- and 2-year follow-up predicted by anhedonia, social anhedonia and outcome at baseline – unadjusted analyses.

|  | | | Level of functioning: Symptoms ^a^ | | | | | | | | | | Level of functioning: Disability | | | | | | | | | | |
| --- | --- | --- | --- | --- | --- | --- | --- | --- | --- | --- | --- | --- | --- | --- | --- | --- | --- | --- | --- | --- | --- | --- | --- |
|  | | | 1-year follow-up *(N=48)* | | | | | 2-year follow-up *(N=36)* | | | | | 1-year follow-up *(N=48)* | | | | 2-year follow-up *(N=36)* | | | | | | |
|  | | | *b* (CI ^b^) | | | *p* | | *b* (CI) | | *p* | | | *b* (CI) | | *p* | | | | *b* (CI) | | | *p* | |
|  | | |  | | |  | |  | |  | | |  | |  | | | |  | | |  | |
| Predictor: Anhedonia | | | | | | | | | | | | | | | | | | | | | | | |
| Outcome at baseline | | | 0.27  (-0.02 – 0.56) | | | .056 | | 0.30  (-0.20 – 0.80) | | .238 | | | 0.38  (0.12 – 0.65) | | .006 | | | | 0.38  (0.00 – 0.76) | | | .052 | |
| Anhedonia | | | 2.73  (-1.02 – 6.48) | | | .150 | | 1.29  (-3.45 – 6.03) | | .585 | | | 5.80  (1.57 – 10.03) | | .008 | | | | 2.06  (-3.22 – 7.34) | | | .433 | |
| Predictor: Social anhedonia | | | | | | | | | | | | | | | | | | | | | | | |
| Outcome at baseline | | | 0.27  (-0.02 – 0.55) | | | .064 | | 0.30  (-0.19 – 0.79) | | .226 | | | 0.35  (0.09 – 0.62) | | .009 | | | | 0.36  (0.00 – 0.73) | | | .053 | |
| Social Anhedonia | | | 3.36  (-0.31 – 7.03) | | | .072 | | 2.79  (-1.94 – 7.51) | | .239 | | | 6.59  (2.47 – 10.72) | | .002 | | | | 4.74  (-0.41 – 9.88) | | | .070 | |
|  | | **Illness severity**^c^ | | | | | | | | | **Remission from UHR status** | | | | | | | | | **Transition status** | | | |
|  | | 1-year follow-up *(N=47)* | | | | | 2-year follow-up *(N=37)* | | | | | *(N=54)* | | | | | | *(N=57)* | | | | | |
|  |  | *b* (CI) | | | *p* | | *b* (CI) | | *p* | | | *HR* ^d^ (CI) | | | | *p* | | *HR* (CI) | | | | | *p* |
| Predictor: Anhedonia | | | | | | | | | | | | | | | | | | | | | | | |
| Outcome at  baseline | | 0.62  (0.34 – 0.89) | | <.001 | | | 0.36  (0.01 – 0.71) | | .044 | | |  | |  | |  | |  | | |  | | |
| Anhedonia | | -0.30  (-0.69 – 0.09) | | .0123 | | | -0.37  (-0.85 – 0.10) | | .121 | | | 2.40  (1.09 – 5.27) | | | | .029 | | 0.67  (0.29 – 1.54) | | | | | .343 |
| Predictor: Social anhedonia | | | | | | | | | | | | | | | | | | | | | | | |
| Outcome at  baseline | | 0.63  (0.36 – 0.89) | | <.001 | | | 0.34  (0.01 – 0.66) | | .044 | | |  | |  | |  | |  | | |  | |  |
| Social Anhedonia | | -0.32  (-0.70 – 0.05) | | .091 | | | -0.56  (-1.02– -0.11) | | .017 | | | 2.08  (1.01– 4.28) | | | | .046 | | 0.75  (0.32 – 1.76) | | | | | .507 |

^a^ Level of functioning assessed with the Global Assessment of Functioning Scale.

^b^ CI, confidence interval.

^c^ Symptom severity assessed with the Clinical Global Impression Scale.

^d^ HR, Hazard ratio.
