# Supplementary material for: Momentary Manifestations of Negative Symptoms as Predictors of Clinical Outcomes in People at High Risk for Psychosis: Experience Sampling Study
Source: JMIR Ment Health. 2021 Nov 19;8(11):e30309. doi: 10.2196/30309 (PMC8663470; doi:10.2196/30309)
Supplement: Multimedia Appendix 4 [file mental_v8i11e30309_app4.docx]

# Supplementary Material 4

## Sensitivity analysis with current depressive episode as an additional independent variable to control for potential confounding

Table S6. Clinical outcomes at 1- and 2-year follow-up predicted by blunted affective experience at baseline (i.e., intensity, instability and variability of negative and positive affect) and clinical outcome at baseline. ^a^

|  | | | | | **Level of functioning: Symptoms** ^b^ | | | | | | | | | **Level of functioning: Disability** | | | | |  |
| --- | --- | --- | --- | --- | --- | --- | --- | --- | --- | --- | --- | --- | --- | --- | --- | --- | --- | --- | --- |
|  | | | | | 1-year follow-up *(N=48)* | | | | 2-year follow-up *(N=36)* | | | | | 1-year follow-up *(N=48)* | | | 2-year follow-up *(N=36)* | |  |
|  | | | | | *b* (CI^c^) | | *p* | | *b* (CI) | | | *p* | | *b* (CI) | | *p* | *b* (CI) | *p* |  |
|  | | | | |  | |  | |  | | |  | |  | |  |  |  |  |
| **Predictor: Intensity NA** | | | | | | | | | | | | | | | | | | | |
| Outcome at baseline | | | | | 0.15  (-0.21 – 0.51) | | .411 | | 0.05  (-0.60 – 0.70) | | | .865 | | 0.30  (-0.05 – 0.65) | | .091 | 0.56  (0.05 – 1.07) | .033 |  |
| Intensity NA^d^ | | | | | -2.18  (-6.46 – 2.10) | | .309 | | -1.67  (-7.37 – 4.04) | | | .553 | | -1.73  (-6.54– 3.08) | | .471 | 1.17  (-5.19 –7.54) | .708 |  |
| **Predictor: Intensity PA** | | | | | | | | | | | | | | | | | | | |
| Outcome at baseline | | | | | 0.14  (-0.21 – 0.50) | | .422 | | 0.05  (-0.59 – 0.69) | | | .869 | | 0.30  (-0.04 – 0.65) | | .079 | 0.57  (0.05 – 1.08) | .031 |  |
| Intensity PA^e^ | | | | | 3.70  (-0.59 – 7.99) | | .089 | | 1.74  (-4.34 – 7.82) | | | .561 | | 3.68  (-1.15 – 8.50) | | .131 | 0.30  (-6.48 – 7.09) | .927 |  |
| **Predictor: Instability NA** | | | | | | | | | | | | | | | | | | | |
| Outcome at baseline | | | | | 0.18  (-0.19 – 0.54) | | .337 | | 0.05  (-0.58 – 0.68) | | | .869 | | 0.31  (-0.05 – 0.66) | | .089 | 0.59  (0.11 – 1.07) | .018 |  |
| Instability NA | | | | | 0.91  (-1.33 – 3.16) | | .417 | | -2.43  (-6.98 – 2.13) | | | .283 | | -0.01  (-2.55 – 2.52) | | .991 | -4.44  (-9.32 – 0.45) | .073 |  |
| **Predictor: Instability PA** | | | | | | | | | | | | | | | | | | | |
| Outcome at baseline | | | | | 0.16  (-0.21 – 0.53) | | .400 | | -0.01  (-0.62 – 0.60) | | | .973 | | 0.30  (-0.05– 0.65) | | .090 | 0.57  (0.12 – 1.03) | .016 |  |
| Instability PA | | | | | -0.45  (-4.01– 3.11) | | .801 | | -4.81  (-10.65 – 1.03) | | | .102 | | -0.88  (-4.82 – 3.06) | | .652 | -7.66  (-13.80 – -1.52) | .017 |  |
| **Predictor: Variability NA** | | | | | | | | | | | | | | | | | | | |
| Outcome at baseline | | | | | 0.16  (-0.21 – 0.52) | | .384 | | 0.09  (-0.54 – 0.72) | | | .765 | | 0.31  (-0.04 – 0.66) | | .084 | 0.56  (0.10 – 1.03) | .020 |  |
| Variability NA | | | | | 2.15  (-2.87 – 7.17) | | .391 | | -5.23  (-13.56 – 3.09) | | | .207 | | 1.37  (-4.29– 7.02) | | .627 | -9.32  (-18.01 – -0.63) | .037 |  |
| **Predictor: Variability PA** | | | | | | | | | | | | | | | | | | | |
| Outcome at baseline | | | | | 0.16  (-0.21 – 0.52) | | .397 | | 0.07  (-0.54 – 0.68) | | | .816 | | 0.31  (-0.04 – 0.66) | | .083 | 0.48  (-0.02 – 0.98) | .057 |  |
| Variability PA | | | | | 1.00  (-4.69 – 6.70) | | .724 | | -5.59  (-12.66 – 1.48) | | | .116 | | 1.58  (-4.74 – 7.90) | | .615 | -6.27  (-14.38 – 1.84) | .124 |  |
|  | | **Illness severity** ^f^ | | | | | | | | | | | **Remission from  UHR status** | | | | **Transition status** | | |
|  | | 1-year follow-up *(N=47)* | | | | | | 2-year follow-up *(N=37)* | | | | | *(N=54)* | | | | *(N=57)* | | |
|  |  | *b* (CI) | | | | *p* | | *b* (CI) | | *p* | | | *HR^g^* (CI) | | *p* | | *HR* (CI) | *p* | |
|  |  |  | | | |  | |  | |  | | |  | |  | |  |  | |
| **Predictor: Intensity NA** | | | | | | | | | | | | | | | | | | | |
| Outcome at  baseline | | 0.40  (0.08 – 0.73) | | | | .017 | | 0.27  (-0.23 – 0.78) | | | .276 | |  | |  | |  | | |
| Intensity NA | | 0.29  (-0.13 – 0.70) | | | | .167 | | -0.03  (-0.61 –0.535 | | | .911 | | 0.28  (0.09 – 0.89) | | .031 | | 1.44  (0.67 – 3.07) | .347 | |
| **Predictor: Intensity PA** | | | | | | | | | | | | | | | | | | | |
| Outcome at  baseline | | 0.42  (0.09 – 0.74) | | | | .013 | | 0.19  (-0.31 – 0.69) | | | .447 | |  | |  | |  |  | |
| Intensity PA | | -0.28  (-0.69 – 0.13) | | | | .176 | | -0.37  (-1.03 – 0.29) | | | .262 | | 2.08  (0.88 – 4.94) | | .096 | | 0.44  (0.15 – 1.33) | .146 | |
| **Predictor: Instability NA** | | | | | | | | | | | | | | | | | | | |
| Outcome at  baseline | | 0.45  (0.12 – 0.78) | | | | .009 | | 0.26  (-0.23 – 0.76) | | | .285 | |  | |  | |  | | |
| Instability NA | | -0.03  (-0.24– 0.17) | | | | .744 | | -0.02  (-0.49 – 0.45) | | | .936 | | 1.21  (0.58 – 2.55) | | .611 | | 1.04  (0.68 – 1.59) | .857 | |
| **Predictor: Instability PA** | | | | | | | | | | | | | | | | | | | |
| Outcome at  baseline | | | 0.45  (0.12 – 0.78) | | | .009 | | 0.29  (-0.20 – 0.78) | | | .237 | |  | |  | |  | | |
| Instability PA | | | -0.05  (-0.37 – 0.28) | | | .775 | | 0.25  (-0.39 – 0.88) | | | .434 | | 1.77  (0.70 – 4.48) | | .230 | | 0.82  (0.33 – 2.04) | .667 | |
| **Predictor: Variability NA** | | | | | | | | | | | | | | | | | | | |
| Outcome at  baseline | | | | 0.44  (0.12 – 0.77) | | .009 | | 0.25  (-0.27 – 0.77) | | | .327 | |  | |  | |  | | |
| Variability NA | | | | -0.14  (-0.58 – 0.30) | | .519 | | -0.09  (-0.97 – 0.79) | | | .839 | | 1.28  (0.29 – 5.57) | | .741 | | 1.37  (0.61 – 3.09) | .447 | |
| **Predictor: Variability PA** | | | | | | | | | | | | | | | | | | | |
| Outcome at baseline | | | | 0.45  (0.12 – 0.78) | | .010 | | 0.38  (-0.13 – 0.89) | | | .138 | |  | |  | |  | | |
| Variability PA | | | | 0.04  (-0.46 – 0.55) | | .860 | | 0.50  (-0.30 – 1.30) | | | .212 | | 4.85  (1.59 – 14.81) | | .006 | | 1.29  (0.42 – 3.99) | .654 | |

*^a^* Results adjusted for age, gender, ethnicity, center, time to follow-up and current depressive episode.

^b^ Level of functioning assessed with the Global Assessment of Functioning Scale.

^c^ CI, confidence interval

^d^ NA, negative affect.

^e^ PA, positive affect.

^f^ Illness severity assessed with the Clinical Global Impression Scale.

^g^ HR, Hazard ratio

Table S7. Clinical outcomes at 1- and 2-year follow-up predicted by lack of social drive (i.e., amount of time spent alone, preference to be alone when in company and experienced pleasantness of being alone) and clinical outcome at baseline. ^a^

|  | | | **Level of functioning: Symptoms** ^b^ | | | | | | | | **Level of functioning: Disability** | | | | | | | | | |  |
| --- | --- | --- | --- | --- | --- | --- | --- | --- | --- | --- | --- | --- | --- | --- | --- | --- | --- | --- | --- | --- | --- |
|  | | | 1-year follow-up *(N=48)* | | | | 2-year follow-up *(N=36)* | | | | 1-year follow-up *(N=48)* | | | | | 2-year follow-up *(N=36)* | | | | |  |
|  | | | *b* (CI ^c^) | | *p* | | *b* (CI) | | *p* | | *b* (CI) | | | | *p* | *b* (CI) | | | *p* | |  |
|  | | |  | |  | |  | |  | |  | | | |  |  | | |  | |  |
| **Predictor: Amount of time spent alone** | | | | | | | | | | | | | | | | | | | | | |
| Outcome at baseline | | | 0.16  (-0.21 – 0.53) | | .378 | | -0.12  (-0.76 – 0.53) | | .714 | | 0.30  (-0.05 – 0.65) | | | | .086 | 0.47  (-0.02 – 0.96) | | | .061 | |  |
| Amount of time spent alone | | | 2.03  (-11.94 – 15.99) | | .771 | | 14.02  (-5.64 – 33.68) | | .154 | | 5.41  (-10.11 – 20.91) | | | | .485 | 19.48  (-1.49 – 40.45) | | | .067 | |  |
| **Predictor: Preference to be alone when in company** | | | | | | | | | | | | | | | | | | | | | |
| Outcome at baseline | | | 0.17  (-0.19 – 0.53) | | .347 | | 0.10  (-0.56 – 0.77) | | .755 | | 0.31  (-0.04 – 0.66) | | | | .080 | 0.55  (0.04 – 1.07) | | | .034 | |  |
| Preference to be alone | | | -1.40  (-4.15 – 1.34) | | .307 | | -1.54  (-5.66 – 2.58) | | .448 | | -0.97  (-4.07 – 2.13) | | | | .529 | -1.13  (-5.56 – 3.30) | | | .604 | |  |
| **Predictor: Pleasantness of being alone** | | | | | | | | | | | | | | | | | | | | | |
| Outcome at baseline | | | 0.16  (-0.21 – 0.54) | | .391 | | 0.17  (-0.46 – 0.79) | | .558 | | 0.34  (-0.03 – 0.70) | | | | .070 | 0.53  (0.08 – 0.98) | | | .023 | |  |
| Pleasantness of being alone | | | 0.17  (-2.78 – 3.11) | | .909 | | -3.18  (-6.76 – 0.40) | | .079 | | -0.99  (-4.33 – 2.34) | | | | .550 | -4.74  (-8.39 – -1.09) | | | .013 | |  |
|  | | **Illness severity** ^d^ | | | | | | | | **Remission from UHR status** | | | | | | | **Transition status** | | | | |
|  | | 1-year follow-up *(N=47)* | | | | 2-year follow-up *(N=37)* | | | | *(N=54)* | | | | | | | *(N=57)* | | | | |
|  |  | *b* (CI) | | *p* | | *b* (CI) | | *p* | | *HR* ^e^ (CI) | | | | *p* | | | *HR* (CI) | | | *p* | |
| **Predictor: Amount of time spent alone** | | | | | | | | | | | | | | | | | | | | | |
| Outcome at  baseline | | 0.41  (0.06 – 0.75) | | .023 | | 0.14  (-0.36 – 0.64) | | .571 | |  | |  | |  | |  | | | |  | |
| Amount of time spent alone | | -0.44  (-1.77 – 0.90) | | .512 | | -1.54  (-3.67 – 0.58) | | .147 | | 5.58  (0.28 – 111.20) | | | | .260 | | | 0.05  (0.00 – 2.07) | | | .116 | |
| **Predictor: Preference to be alone when in company** | | | | | | | | | | | | | | | | | | | | | |
| Outcome at  baseline | | 0.43  (0.11 – 0.76) | | .011 | | 0.24  (-0.24 – 0.73) | | .310 | |  | | |  |  | |  | |  | |  | |
| Preference to be alone | | 0.09  (-0.17 – 0.35) | | .505 | | 0.23  (-0.17 – 0.64) | | .252 | | 0.97  (0.51 – 1.86) | | | | .929 | | | 1.17  (0.61 – 2.23) | | | .635 | |
| **Predictor: Pleasantness of being alone** | | | | | | | | | | | | | | | | | | | | | |
| Outcome at  baseline | | 0.45  (0.11 – 0.78) | | .011 | | 0.34  (-0.16 – 0.83) | | .179 | |  | | |  |  | |  | |  | | | |
| Pleasantness of being alone | | 0.04  (-0.21 – 0.29) | | .730 | | 0.19  (-0.17 – 0.56) | | .285 | | 0.82  (0.44 – 1.54) | | | | .540 | | | 1.47  (0.76 – 2.87) | | | .255 | |

^a^ Results adjusted for age, gender, ethnicity, center, time to follow-up and current depressive episode.

^b^ Level of functioning assessed with the Global Assessment of Functioning Scale.

^c^ CI, confidence interval

^d^ Illness severity assessed with the Clinical Global Impression Scale.

^e^ HR, Hazard ratio.

Table S8. Clinical Outcomes at 1- and 2-year follow-up predicted by anhedonia, social anhedonia and clinical outcome at baseline. ^a^

|  | | | **Level of functioning: Symptoms** ^b^ | | | | | | | | | | **Level of functioning: Disability** | | | | | | | | |  |
| --- | --- | --- | --- | --- | --- | --- | --- | --- | --- | --- | --- | --- | --- | --- | --- | --- | --- | --- | --- | --- | --- | --- |
|  | | | 1-year follow-up *(N=48)* | | | | | | 2-year follow-up *(N=36)* | | | | 1-year follow-up *(N=48)* | | | | 2-year follow-up *(N=36)* | | | | |  |
|  | | | *b* (CI ^c^) | | | *p* | | | *b* (CI) | | *p* | | *b* (CI) | | *p* | | | | *b* (CI) | *p* | |  |
|  | | |  | | |  | | |  | |  | |  | |  | | | |  |  | |  |
| **Predictor: Anhedonia** | | | | | | | | | | | | | | | | | | | | | | |
| Outcome at baseline | | | 0.15  (-0.17 – 0.49) | | | .394 | | | -0.04  (-0.61 – 0.68) | | .907 | | 0.30  (-0.03 – 0.64) | | .076 | | | | 0.57  (0.05 – 1.08) | .031 | |  |
| Anhedonia events | | | 3.56  (-0.86 – 7.98) | | | .111 | | | 0.78  (-5.24 – 6.80) | | .791 | | 4.31  (-0.59 – 9.21) | | .083 | | | | -0.22  (-6.90 – 6.46) | .946 | |  |
| **Predictor: Social anhedonia** | | | | | | | | | | | | | | | | | | | | | | |
| Outcome at baseline | | | 0.16  (-0.19 – 0.50) | | | .362 | | | 0.08  (-0.56 – 0.72) | | .807 | | 0.30  (-0.03 – 0.62) | | .074 | | | | 0.54  (0.04 – 1.05) | .035 | |  |
| Social Anhedonia | | | 4.47  (0.38 – 8.56) | | | .033 | | | 2.80  (-3.38 – 8.98) | | .359 | | 5.35  (0.83 – 9.88) | | .022 | | | | 3.34  (-3.49 – 10.16) | .324 | |  |
|  | | **Illness severity** ^d^ | | | | | | | | | | **Remission from UHR status** | | | | | | **Transition status** | | | | |
|  | | 1-year follow-up *(N=47)* | | | | | 2-year follow-up *(N=37)* | | | | | *(N=54)* | | | | | | *(N=57)* | | | | |
|  |  | *b* (CI) | | | *p* | | | *b* (CI) | | *p* | | *HR* (CI) | | | | *p* | | *HR* (CI) | | | *p* | |
| **Predictor: Anhedonia** | | | | | | | | | | | | | | | | | | | | | | |
| Outcome at  baseline | | 0.42  (0.09 – 0.74) | | .013 | | | | 0.20  (-0.30 – 0.71) | | .415 | |  | |  | |  | |  | | |  | |
| Anhedonia | | -0.26  (-0.68 – 0.15) | | .206 | | | | -0.31  (-0.96 – 0.34) | | .334 | | 2.02  (0.82 – 4.97) | | | | .125 | | 0.49  (0.16 – 1.52) | | | .217 | |
| **Predictor: Social anhedonia** | | | | | | | | | | | | | | | | | | | | | | |
| Outcome at  baseline | | 0.41  (0.09 – 0.72) | | .013 | | | | 0.16  (-0.32 – 0.64) | | .505 | |  | |  | |  | |  | | |  | |
| Social Anhedonia | | -0.35  (-0.73 – 0.03) | | .069 | | | | -0.58  (-1.21 – 0.05) | | .072 | | 2.26  (0.84 – 6.04) | | | | .105 | | 0.57  (0.21 – 1.59) | | | .286 | |

^a^ Results adjusted for age, gender, ethnicity, center, time to follow-up and current depressive episode.

^b^ Level of functioning assessed with the Global Assessment of Functioning Scale.

^c^ CI, confidence interval*.*

^d^ Illness severity assessed with the Clinical Global Impression Scale. HR, Hazard ratio;
