# Supplementary material for: Momentary Manifestations of Negative Symptoms as Predictors of Clinical Outcomes in People at High Risk for Psychosis: Experience Sampling Study
Source: JMIR Ment Health. 2021 Nov 19;8(11):e30309. doi: 10.2196/30309 (PMC8663470; doi:10.2196/30309)
Supplement: Multimedia Appendix 5 [file mental_v8i11e30309_app5.docx]

# Supplementary Material 5

## Sensitivity analysis with comorbid Axis-I disorder as an additional independent variable to control for potential confounding

Table S9. Clinical outcomes at 1- and 2-year follow-up predicted by blunted affective experience at baseline (i.e., intensity, instability and variability of negative and positive affect) and clinical outcome at baseline. ^a^

|  | | | | | **Level of functioning: Symptoms** ^b^ | | | | | | | | **Level of functioning: Disability** | | | | |  |
| --- | --- | --- | --- | --- | --- | --- | --- | --- | --- | --- | --- | --- | --- | --- | --- | --- | --- | --- |
|  | | | | | 1-year follow-up *(N=48)* | | | | 2-year follow-up *(N=36)* | | | | 1-year follow-up *(N=48)* | | | 2-year follow-up *(N=36)* | |  |
|  | | | | | *b* (CI^c^) | | *p* | | *b* (CI) | | *p* | | *b* (CI) | | *p* | *b* (CI) | *p* |  |
|  | | | | |  | |  | |  | |  | |  | |  |  |  |  |
| **Predictor: Intensity NA** | | | | | | | | | | | | | | | | | | |
| Outcome at baseline | | | | | 0.21  (-0.15 – 0.57) | | .247 | | 0.15  (-0.52 – 0.82) | | .647 | | 0.35  (-0.01 – 0.71) | | .059 | 0.55  (0.06 – 1.04) | .029 |  |
| Intensity NA^d^ | | | | | -2.43  (-6.51 – 1.65) | | .236 | | -1.75  (-7.33 – 3.82) | | .523 | | -3.17  (-7.88 – 1.55) | | .181 | 1.01  (-5.13 –7.15) | .738 |  |
| **Predictor: Intensity PA** | | | | | | | | | | | | | | | | | | |
| Outcome at baseline | | | | | 0.20  (-0.15 – 0.54) | | .254 | | 0.18  (-0.50 – 0.85) | | .594 | | 0.34  (0.00 – 0.69) | | .049 | 0.55  (0.06 – 1.03) | .029 |  |
| Intensity PA^e^ | | | | | 4.12  (0.14 – 8.10) | | .043 | | 2.45  (-3.62 – 8.52) | | .413 | | 5.38  (0.81 – 9.96) | | .022 | 1.26  (-5.37 – 7.90) | .699 |  |
| **Predictor: Instability NA** | | | | | | | | | | | | | | | | | | |
| Outcome at baseline | | | | | 0.31  (-0.06 – 0.68) | | .099 | | 0.09  (-0.58 – 0.76) | | .787 | | 0.37  (-0.01 – 0.74) | | .053 | 0.55  (0.07 – 1.02) | .025 |  |
| Instability NA | | | | | 1.32  (-1.13 – 3.76) | | .283 | | -1.14  (-5.66 – 3.38) | | .608 | | -0.27  (-3.12– 2.59) | | .851 | -3.12  (-7.92 – 1.69) | .193 |  |
| **Predictor: Instability PA** | | | | | | | | | | | | | | | | | | |
| Outcome at baseline | | | | | 0.26  (-0.11 – 0.63) | | .166 | | 0.00  (-0.66 – 0.67) | | .992 | | 0.37  (0.00 – 0.74) | | .049 | 0.55  (0.10 – 1.00) | .018 |  |
| Instability PA | | | | | 0.18  (-3.62 – 3.99) | | .923 | | -4.15  (-10.56 – 2.26) | | .194 | | -0.32  (-4.71 – 4.06) | | .882 | -7.00  (-13.48 – -0.51) | .036 |  |
| **Predictor: Variability NA** | | | | | | | | | | | | | | | | | | |
| Outcome at baseline | | | | | 0.30  (-0.07 – 0.66) | | .106 | | 0.10  (-0.54 – 0.76) | | .765 | | 0.37  (0.00 – 0.74) | | .047 | 0.53  (0.06 – 0.99) | .028 |  |
| Variability NA | | | | | 2.99  (-2.57 – 8.55) | | .283 | | -2.77  (-11.13 – 5.59) | | .502 | | 0.44  (-6.08 – 6.96) | | .892 | -7.04  (-15.92 – 1.84) | .115 |  |
| **Predictor: Variability PA** | | | | | | | | | | | | | | | | | | |
| Outcome at baseline | | | | | 0.25  (-0.10 – 0.61) | | .159 | | 0.09  (-0.55 – 0.74) | | .769 | | 0.38  (0.01 – 0.74) | | .043 | 0.49  (0.00 – 0.97) | .050 |  |
| Variability PA | | | | | 2.12  (-4.05 – 8.30) | | .490 | | -4.89  (-12.40 – 2.63) | | .192 | | 3.09  (-4.17 – 10.35) | | .394 | -5.26  (-13.82 – 3.31) | .218 |  |
|  | | **Illness severity** ^f^ | | | | | | | | | | **Remission from  UHR status** | | | | **Transition status** | | |
|  | | 1-year follow-up *(N=47)* | | | | | | 2-year follow-up *(N=37)* | | | | *(N=54)* | | | | *(N=57)* | | |
|  |  | *b* (CI) | | | | *p* | | *b* (CI) | | *p* | | *HR^g^* (CI) | | *p* | | *HR* (CI) | *p* | |
|  |  |  | | | |  | |  | |  | |  | |  | |  |  | |
| **Predictor: Intensity NA** | | | | | | | | | | | | | | | | | | |
| Outcome at  baseline | | 0.45  (0.12 – 0.78) | | | | .008 | | 0.36  (-0.18 – 0.89) | | .181 | |  | |  | |  | | |
| Intensity NA | | 0.31  (-0.10 – 0.71) | | | | .133 | | -0.06  (-0.64 –0.52) | | .831 | | 0.31  (0.11 – 0.86) | | .025 | | 1.44  (0.66 – 3.15) | .364 | |
| **Predictor: Intensity PA** | | | | | | | | | | | | | | | | | | |
| Outcome at  baseline | | 0.48  (0.17 – 0.79) | | | | .004 | | 0.25  (-0.27 – 0.77) | | .337 | |  | |  | |  |  | |
| Intensity PA | | -0.32  (-0.70 – 0.07) | | | | .101 | | -0.37  (-1.00 –0.27) | | .247 | | 2.73  (1.02 – 7.33) | | .046 | | 0.60  (0.22 – 1.66) | .324 | |
| **Predictor: Instability NA** | | | | | | | | | | | | | | | | | | |
| Outcome at  baseline | | 0.56  (0.23 – 0.89) | | | | .001 | | 0.34  (-0.17 – 0.85) | | .178 | |  | |  | |  | | |
| Instability NA | | -0.05  (-0.27 – 0.16) | | | | .631 | | -0.04  (-0.48 – 0.41) | | .873 | | 1.25  (0.50 – 3.17) | | .639 | | 1.01  (0.65 – 1.56) | .982 | |
| **Predictor: Instability PA** | | | | | | | | | | | | | | | | | | |
| Outcome at  baseline | | | 0.56  (0.24 – 0.87) | | | .001 | | 0.33  (-0.17 – 0.84) | | .184 | |  | |  | |  | | |
| Instability PA | | | -0.11  (-0.45 – 0.24) | | | .535 | | 0.21  (-0.44 – 0.86) | | .520 | | 1.89  (0.62 – 5.68) | | .257 | | 0.95  (0.46 – 1.98) | .899 | |
| **Predictor: Variability NA** | | | | | | | | | | | | | | | | | | |
| Outcome at  baseline | | | | 0.56  (0.25 – 0.88) | | .001 | | 0.33  (-0.18 – 0.84) | | .191 | |  | |  | |  | | |
| Variability NA | | | | -0.18  (-0.65 – 0.29) | | .447 | | -0.12  (-0.96 – 0.71) | | .763 | | 1.28  (0.24 – 6.94) | | .773 | | 1.19  (0.52 – 2.74) | .679 | |
| **Predictor: Variability PA** | | | | | | | | | | | | | | | | | | |
| Outcome at baseline | | | | 0.54  (0.22 – 0.86) | | .001 | | 0.41  (-0.10 – 0.92) | | .110 | |  | |  | |  | | |
| Variability PA | | | | -0.02  (-0.57 – 0.53) | | .944 | | 0.46  (-0.35 – 1.27) | | .252 | | 9.13  (2.02 – 41.30) | | .004 | | 1.48  (0.49 – 4.45) | .489 | |

*^a^* Results adjusted for age, gender, ethnicity, center, time to follow-up and comorbid disorders.

^b^ Level of functioning assessed with the Global Assessment of Functioning Scale.

^c^ CI, confidence interval

^d^ NA, negative affect.

^e^ PA, positive affect.

^f^ Illness severity assessed with the Clinical Global Impression Scale.

^g^ HR, Hazard ratio

Table S10. Clinical outcomes at 1- and 2-year follow-up predicted by lack of social drive (i.e., amount of time spent alone, preference to be alone when in company and experienced pleasantness of being alone) and clinical outcome at baseline. ^a^

|  | | | **Level of functioning: Symptoms** ^b^ | | | | | | | | **Level of functioning: Disability** | | | | | | | | | |  |
| --- | --- | --- | --- | --- | --- | --- | --- | --- | --- | --- | --- | --- | --- | --- | --- | --- | --- | --- | --- | --- | --- |
|  | | | 1-year follow-up *(N=48)* | | | | 2-year follow-up *(N=36)* | | | | 1-year follow-up *(N=48)* | | | | | 2-year follow-up *(N=36)* | | | | |  |
|  | | | *b* (CI ^c^) | | *p* | | *b* (CI) | | *p* | | *b* (CI) | | | | *p* | *b* (CI) | | | *p* | |  |
|  | | |  | |  | |  | |  | |  | | | |  |  | | |  | |  |
| **Predictor: Amount of time spent alone** | | | | | | | | | | | | | | | | | | | | | |
| Outcome at baseline | | | 0.26  (-0.10 – 0.62) | | .154 | | 0.07  (-0.56 – 0.69) | | .832 | | 0.37  (0.01 – 0.74) | | | | .046 | 0.47  (0.03 – 0.92) | | | .037 | |  |
| Amount of time spent alone | | | 2.80  (-11.56 – 17.51) | | .695 | | 16.24  (-1.43 – 33.91) | | .070 | | 5.17  (-11.65 – 21.99) | | | | .537 | 22.59  (3.66 – 41.52) | | | .021 | |  |
| **Predictor: Preference to be alone when in company** | | | | | | | | | | | | | | | | | | | | | |
| Outcome at baseline | | | 0.24  (-0.11 – 0.59) | | .172 | | 0.29  (-0.43 – 1.00) | | .419 | | 0.37  (0.01 – 0.73) | | | | .043 | 0.53  (0.05 – 1.02) | | | .032 | |  |
| Preference to be alone | | | -1.69  (-4.30 – 0.92) | | .197 | | -2.41  (-6.66 – 1.84) | | .254 | | -1.94  (-5.02 – 1.14) | | | | .210 | -1.66  (-6.03 – 2.70) | | | .440 | |  |
| **Predictor: Pleasantness of being alone** | | | | | | | | | | | | | | | | | | | | | |
| Outcome at baseline | | | 0.25  (-0.11 – 0.62) | | .169 | | 0.17  (-0.47 – 0.82) | | .585 | | 0.41  (0.03 – 0.79) | | | | .035 | 0.51  (0.07 – 0.95) | | | .026 | |  |
| Pleasantness of being alone | | | 0.15  (-2.82 – 3.12) | | .918 | | -2.62  (-6.21 – 0.97) | | .145 | | -1.36  (-4.93 – 2.21) | | | | .445 | -4.30  (-8.04 – -0.55) | | | .026 | |  |
|  | | **Illness severity** ^d^ | | | | | | | | **Remission from UHR status** | | | | | | | **Transition status** | | | | |
|  | | 1-year follow-up *(N=47)* | | | | 2-year follow-up *(N=37)* | | | | *(N=54)* | | | | | | | *(N=57)* | | | | |
|  |  | *b* (CI) | | *p* | | *b* (CI) | | *p* | | *HR* ^e^ (CI) | | | | *p* | | | *HR* (CI) | | | *p* | |
| **Predictor: Amount of time spent alone** | | | | | | | | | | | | | | | | | | | | | |
| Outcome at  baseline | | 0.52  (0.20 – 0.84) | | .002 | | 0.32  (-0.16 – 0.81) | | .185 | |  | |  | |  | |  | | | |  | |
| Amount of time spent alone | | -0.35  (-1.68 – 0.98) | | .600 | | -1.35  (-3.23 – 0.53) | | .152 | | 4.35  (0.32 – 59.89) | | | | .272 | | | 0.02  (0.00 – 1.12) | | | .056 | |
| **Predictor: Preference to be alone when in company** | | | | | | | | | | | | | | | | | | | | | |
| Outcome at  baseline | | 0.51  (0.19 – 0.83) | | .002 | | 0.32  (-0.17 – 0.81) | | .192 | |  | | |  |  | |  | |  | |  | |
| Preference to be alone | | 0.11  (-0.14 – 0.36) | | .368 | | 0.24  (-0.16 – 0.64) | | .226 | | 0.85  (0.43 – 1.67) | | | | .632 | | | 1.21  (0.64 – 2.27) | | | .555 | |
| **Predictor: Pleasantness of being alone** | | | | | | | | | | | | | | | | | | | | | |
| Outcome at  baseline | | 0.54  (0.22 – 0.86) | | .002 | | 0.37  (-0.13 – 0.87) | | .139 | |  | | |  |  | |  | |  | | | |
| Pleasantness of being alone | | 0.05  (-0.20 – 0.29) | | .715 | | 0.18  (-0.19 – 0.54) | | .329 | | 0.89  (0.48 – 1.66) | | | | .716 | | | 1.38  (0.73 – 2.59) | | | .316 | |

^a^ Results adjusted for age, gender, ethnicity, center, time to follow-up and comorbid disorders.

^b^ Level of functioning assessed with the Global Assessment of Functioning Scale.

^c^ CI, confidence interval

^d^ Illness severity assessed with the Clinical Global Impression Scale.

^e^ HR, Hazard ratio.

Table S11. Clinical Outcomes at 1- and 2-year follow-up predicted by anhedonia, social anhedonia and clinical outcome at baseline. ^a^

|  | | | **Level of functioning: Symptoms** ^b^ | | | | | | | | | | **Level of functioning: Disability** | | | | | | | | |  |
| --- | --- | --- | --- | --- | --- | --- | --- | --- | --- | --- | --- | --- | --- | --- | --- | --- | --- | --- | --- | --- | --- | --- |
|  | | | 1-year follow-up *(N=48)* | | | | | | 2-year follow-up *(N=36)* | | | | 1-year follow-up *(N=48)* | | | | 2-year follow-up *(N=36)* | | | | |  |
|  | | | *b* (CI ^c^) | | | *p* | | | *b* (CI) | | *p* | | *b* (CI) | | *p* | | | *b* (CI) | | *p* | |  |
|  | | |  | | |  | | |  | |  | |  | |  | | |  | |  | |  |
| **Predictor: Anhedonia** | | | | | | | | | | | | | | | | | | | | | | |
| Outcome at baseline | | | 0.21  (-0.13 – 0.56) | | | .221 | | | 0.15  (-0.53 – 0.84) | | .645 | | 0.34  (0.00 – 0.68) | | .047 | | | 0.55  (0.06 – 1.04) | | .029 | |  |
| Anhedonia events | | | 4.12  (-0.01 – 8.24) | | | .050 | | | 1.46  (-4.56 – 7.48) | | .621 | | 6.08  (1.40 – 10.75) | | .012 | | | 0.75  (-5.79 – 7.29) | | .815 | |  |
| **Predictor: Social anhedonia** | | | | | | | | | | | | | | | | | | | | | | |
| Outcome at baseline | | | 0.22  (-0.11 – 0.55) | | | .186 | | | 0.24  (-0.43 – 0.91) | | .471 | | 0.334  (0.01 – 0.67) | | .045 | | | 0.51  (0.03 – 0.98) | | .037 | |  |
| Social Anhedonia | | | 4.90  (0.98 – 8.91) | | | .016 | | | 4.05  (-2.27 – 10.36) | | .199 | | 6.67  (2.19 – 11.14) | | .005 | | | 4.70  (-2.02 – 11.44) | | .162 | |  |
|  | | **Illness severity** ^d^ | | | | | | | | | | **Remission from UHR status** | | | | | | **Transition status** | | | | |
|  | | 1-year follow-up *(N=47)* | | | | | 2-year follow-up *(N=37)* | | | | | *(N=54)* | | | | | | *(N=57)* | | | | |
|  |  | *b* (CI) | | | *p* | | | *b* (CI) | | *p* | | *HR* ^e^ (CI) | | | | *p* | | *HR* (CI) | | | *p* | |
| **Predictor: Anhedonia** | | | | | | | | | | | | | | | | | | | | | | |
| Outcome at  baseline | | 0.48  (0.17 – 0.80) | | .004 | | | | 0.26  (-0.25 – 0.78) | | .305 | |  | |  | |  | |  |  | | | |
| Anhedonia | | -0.32  (-0.71 – 0.08) | | .116 | | | | -0.32  (-0.95 – 0.31) | | .305 | | 2.68  (0.94 – 7.60) | | | | .064 | | 0.64  (0.22 – 1.89) | | | .418 | |
| **Predictor: Social anhedonia** | | | | | | | | | | | | | | | | | | | | | | |
| Outcome at  baseline | | 0.48  (0.17 – 0.78) | | .003 | | | | 0.23  (-0.25 – 0.72) | | .338 | |  | |  | |  | |  |  | |  | |
| Social Anhedonia | | -0.39  (-0.76 – -0.02) | | .039 | | | | -0.61  (-1.23 – 0.02) | | .056 | | 4.71  (1.07 – 20.73) | | | | .041 | | 0.68  (0.25 – 1.83) | | | .449 | |

^a^ Results adjusted for age, gender, ethnicity, center, time to follow-up and comorbid disorders.

^b^ Level of functioning assessed with the Global Assessment of Functioning Scale.

^c^ CI, confidence interval*.*

^d^ Illness severity assessed with the Clinical Global Impression Scale.

^e^ HR, Hazard ratio;
