# Supplementary material for: Momentary Manifestations of Negative Symptoms as Predictors of Clinical Outcomes in People at High Risk for Psychosis: Experience Sampling Study
Source: JMIR Ment Health. 2021 Nov 19;8(11):e30309. doi: 10.2196/30309 (PMC8663470; doi:10.2196/30309)
Supplement: Multimedia Appendix 6 [file mental_v8i11e30309_app6.docx]

# Supplementary Material 6

## Comorbid Axis-I diagnoses at baseline

Table S12. Comorbid Axis-I diagnoses at basline

|  | ESM sample | | | No ESM sample | Comparison  ESM vs. no ESM |
| --- | --- | --- | --- | --- | --- |
|  | Baseline | 1-year  follow-up | 2-year  follow-up | Baseline | Baseline |
| Sample Size *N* | 79 | 48 | 36 | 266 |  |
| Comorbidity at baseline *N*(%) | 60 (76%) | 37 (77%) | 28 (78%) | 220 (83%) | χ^2^=1.82, *P*=.177 |
| Major depressive disorder *N*(%) | 29 (37%) | 14 (31%) | 11 (31%) | 123 (51%) | χ^2^=4.67, *P*=.031 |
| Current depressive episode *N*(%) | 22 (28%) | 11 (24%) | 8 (22%) | 88 (35%) | χ^2^=1.26, *P*=.262 |
| Bipolar disorder *N*(%) | 7 (9%) | 4 (9%) | 5 (14%) | 17 (6%) | χ^2^=0.57, *P*=.449 |
| Any anxiety disorder *N*(%) | 42 (53%) | 26 (57%) | 17 (47%) | 117 (44%) | χ^2^=2.06, *P*=.151 |
| Panic disorder *N*(%) | 19 (24%) | 12 (27%) | 6 (17%) | 52 (21%) | χ^2^=0.30, *P*=.584 |
| Panic disorder + agoraphobia *N*(%) | 6 (8%) | 4 (9%) | 1 (3%) | 25 (11%) | χ^2^=0.46, *P*=.496 |
| Agoraphobia only *N*(%) | 2 (3%) | 0 | 0 | 4 (2%) | χ^2^=0.26, *P*=.607 |
| Social phobia *N*(%) | 19 (24%) | 14 (30%) | 9 (25%) | 42 (17%) | χ^2^=1.87, *P*=.172 |
| Specific phobia *N*(%) | 14 (18%) | 9 (20%) | 5 (14%) | 22 (9%) | χ^2^=4.86, *P*=.027 |
| Generalized anxiety disorder *N*(%) | 11 (14%) | 7 (15%) | 5 (14%) | 26 (11%) | χ^2^=0.67, *P*=.413 |
| Not otherwise specified anxiety disorder *N*(%) | 3 (4%) | 1 (2%) | 0 | 14 (6%) | χ^2^=0.49, *P*=.485 |
| Obsessive-compulsive disorder *N*(%) | 3 (4%) | 2 (4%) | 3 (9%) | 26 (12%) | χ^2^=3.41, *P*=.065 |
| Posttraumatic stress disorder *N*(%) | 11 (14%) | 4 (9%) | 0 | 23 (6%) | χ^2^=1.40, *P*=.237 |
| Any eating disorder *N*(%) | 10 (13%) | 7 (15%) | 6 (17%) | 22 (8%) | χ^2^=1.39, *P*=.238 |
| Anorexia nervosa *N*(%) | 5 (6%) | 3 (7%) | 3 (8%) | 10 (4%) | χ^2^=0.69, *P*=.408 |
| Bulimia nervosa *N*(%) | 5 (6%) | 3 (7%) | 2 (6%) | 10 (4%) | χ^2^=0.66, *P*=.417 |
| Binge eating disorder *N*(%) | 1 (1%) | 1 (2%) | 1 (3%) | 6 (3%) | χ^2^=0.44, *P*=.508 |
| Any somatoform disorder *N*(%) | 2 (3%) | 1 (2%) | 1 (3%) | 9 (3%) | χ^2^=0.14, *P*=.705 |
| Somatization disorder *N*(%) | 1 (1%) | 0 | 0 | 4 (2%) | χ^2^=0.06, *P*=.812 |
| Chronic pain *N*(%) | 1 (1%) | 0 | 0 | 1 (<1%) | χ^2^=0.70, *P*=.403 |
| Hypochondriasis *N*(%) | 1 (1%) | 1 (2%) | 1 (3%) | 4 (2%) | χ^2^=0.07, *P*=.789 |
| Body dismorph disorder *N*(%) | 0 | 0 | 0 | 2 (1%) | χ^2^=0.67, *P*=.412 |
