# Supplementary material for: Momentary Manifestations of Negative Symptoms as Predictors of Clinical Outcomes in People at High Risk for Psychosis: Experience Sampling Study
Source: JMIR Ment Health. 2021 Nov 19;8(11):e30309. doi: 10.2196/30309 (PMC8663470; doi:10.2196/30309)
Supplement: Multimedia Appendix 7 [file mental_v8i11e30309_app7.docx]

# Supplementary Material 7

## Restricted analyses

The restricted sample only comprises participants, who returned within a +/- 6 month time interval around the expected follow-up time points. The analyses were conducted with varying sample sizes for illness severity and level of functioning.

Table S13. Clinical outcomes at 1- and 2-year follow-up predicted by blunted affective experience at baseline (i.e., intensity, instability and variability of negative and positive affect) and clinical outcome at baseline – restricted analyses. ^a^

|  | Illness severity ^b^ | | | | | | Level of functioning: Symptoms ^c^ | | | | Level of functioning: Disability | | | |
| --- | --- | --- | --- | --- | --- | --- | --- | --- | --- | --- | --- | --- | --- | --- |
|  | 1-year follow-up *(N*=42) | | | 2-year follow-up *(N=*32) | | | 1-year follow-up *(N*=46) | | 2-year follow-up *(N*=31) | | 1-year follow-up *(N*=46) | | 2-year follow-up *(N*=31) | |
|  | *b* (CI ^d^) | | *p* | *b* (CI) | | *p* | *b* (CI) | *p* | *b* (CI) | *p* | *b* (CI) | *p* | *b* (CI) | *p* |
|  |  | |  |  | |  |  |  |  |  |  |  |  |  |
| Predictor: Intensity NA ^e^ | | | | | | | | | | | | | | |
| **Outcome at baseline** | 0.57  (0.31 – 0.84) | <.001 | | 0.47  (-0.06 – 1.01) | | .082 | 0.28  (-0.03 – 0.60) | .078 | 0.10  (-0.54 – 0.74) | .745 | 0.41  (0.07 – 0.76) | .021 | 0.57  (0.08 – 1.06) | .024 |
| **Intensity NA** | 0.23  (-0.11 – 0.56) | .174 | | -0.22  (-0.79 – 0.35) | | .439 | -2.68  (-6.41 – 1.05) | .155 | 1.53  (-3.57 – 6.64) | .540 | -2.70  (-7.21 – 1.81) | .233 | 4.22  (-1.80 – 10.23) | .160 |
| Predictor: Intensity PA ^f^ | | | | | | | | | | | | | | |
| **Outcome at baseline** | 0.60  (0.34 – 0.86) | <.001 | | 0.33  (-0.26 – 0.91) | | .260 | 0.29  (-0.02 – 0.62) | .065 | 0.14  (-0.49 – 0.78) | .643 | 0.42  (0.09 – 0.75) | .014 | 0.56  (0.04 – 1.07) | .035 |
| **Intensity PA** | -0.18  (-0.54 – 0.18) | .315 | | -0.23  (-0.90 – 0.44) | | .485 | 3.02  (-0.91 – 6.95) | .128 | 0.60  (-4.47 – 5.67) | .808 | 4.63  (0.02 – 9.25) | .049 | -0.88  (-7.15 – 5.40) | .775 |
|  | | | | |  | | | | | | | | | |
| Predictor: Instability NA | | | | | | | | | | | | | | |
| **Outcome at baseline** | 0.65  (0.40 – 0.90) | <.001 | | 0.42  (-0.11 – 0.94) | | .114 | 0.36  (0.04 – 0.68) | .028 | 0.14  (-0.49 – 0.76) | .657 | 0.43  (0.08 – 0.79) | .018 | 0.56  (0.08 – 1.05) | .024 |
| **Instability NA** | -0.02  (-0.19 – 0.15) | .838 | | -0.05  (-0.49 – 0.39) | | .809 | 0.78  (-1.36 – 2.92) | .464 | -1.06  (-4.75 – 2.64) | .558 | -0.44  (-3.08 – 2.20) | .737 | -3.51  (-7.86 – 0.84) | .109 |
| Predictor: Instability PA | | | | | | | | | | | | | | |
| **Outcome at baseline** | 0.65  (0.40 – 0.90) | <.001 | | 0.43  (-0.09 – 0.96) | | .101 | 0.33  (0.01 – 0.65) | .042 | 0.06  (-0.57 – 0.68) | .851 | 0.44  (0.09 – 0.79) | .015 | 0.55  (0.08 – 1.02) | .023 |
| **Instability PA** | -0.06  (-0.33 – 0.21) | .672 | | 0.15  (-0.49 – 0.79) | | .632 | -0.19  (-3.62 – 3.24) | .910 | -3.10  (-8.44 – 2.24) | .241 | -0.64  (-4.77 – 3.49) | .755 | -6.02  (-12.15 – 0.10) | .053 |
|  | | | | |  | | | | | | | | | |
| Predictor: Variability NA | | | | | | | | | | | | | | |
| **Outcome at baseline** | 0.65  (0.40 – 0.90) | <.001 | | 0.41  (-0.12 – 0.94) | | .121 | 0.36  (0.05 – 0.68) | .026 | 0.14  (-0.47 – 0.76) | .635 | 0.45  (0.09 – 0.81) | .015 | 0.58  (0.11 – 1.04) | .017 |
| **Variability NA** | -0.12  (-0.48 – 0.25) | .514 | | -0.15  (-0.95 – 0.65) | | .706 | 2.05  (-2.64 – 6.74) | .382 | -3.29  (-10.04 – 3.45) | .323 | 0.12  (-5.64 – 5.88) | .967 | -8.14  (-15.92 - -0.37) | .041 |
| Predictor: Variability PA | | | | | | | | | | | | | | |
| **Outcome at baseline** | 0.64  (0.39 – 0.89) | <.001 | | 0.48  (-0.04 – 1.01) | | .071 | 0.34  (0.03 – 0.66) | .031 | 0.09  (-0.49 – 0.68) | .751 | 0.46  (0.12 – 0.81) | .010 | 0.50  (0.02 – 0.99) | .043 |
| **Variability PA** | -0.12  (-0.58 – 0.35) | .606 | | 0.41  (-0.37 – 1.18) | | .289 | 2.93  (-2.89 – 8.75) | .314 | -5.77  (-11.91 – 0.37) | .064 | 3.09  (-4.01 – 10.20) | .384 | -6.20  (-14.01 – 1.60) | .114 |

^a^ Results adjusted for age, gender, ethnicity and centre.

^b^ Symptom severity assessed with the Clinical Global Impression Scale.

^c^ Level of functioning assessed with the Global Assessment of Functioning Scale.

^d^ CI, Confidence interval.

^e^ NA, negative affect.

^f^ PA, positive affect.

Table S14. Clinical Outcomes at 1- and 2-year follow-up predicted by lack of social drive (i.e., amount of time spent alone, preference to be alone when in company and experienced pleasantness of being alone) and clinical outcome at baseline – restricted analyses. ^a^

|  | Illness severity ^b^ | | | | Level of functioning: Symptoms ^c^ | | | | Level of functioning: Disability | | | |
| --- | --- | --- | --- | --- | --- | --- | --- | --- | --- | --- | --- | --- |
|  | 1-year follow-up (*N*=42) | | 2-year follow-up *(N*=32) | | 1-year follow-up *(N*=46) | | 2-year follow-up *(N*=31) | | 1-year follow-up *(N*=46) | | 2-year follow-up *(N*=31) | |
|  | *b* (CI ^d^) | *p* | *b* (CI) | *p* | *b* (CI) | *p* | *b* (CI) | *p* | *b* (CI) | *p* | *b* (CI) | *p* |
|  |  |  |  |  |  |  |  |  |  |  |  |  |
| Predictor: Amount of time spent alone | | | | | | | | | | | | |
| **Outcome at baseline** | 0.69  (0.43 – 0.95) | <.001 | 0.35  (-0.17 – 0.86) | .178 | 0.33  (0.02 – 0.65) | .039 | 0.04  (-0.56 – 0.63) | .900 | 0.44  (0.09 – 0.79) | .014 | 0.46  (-0.02 – 0.94) | .057 |
| **Amount of time spent alone** | 0.65  (-0.57 – 1.87) | .288 | -1.22  (-3.03 – 0.59) | .177 | 0.63  (-12.47 – 13.72) | .923 | 13.74  (-1.04 – 28.52) | .067 | 5.18  (-10.66 – 21.03) | .512 | 17.72  (-0.52 – 35.95) | .056 |
| Predictor: Preference to be alone when in company | | | | | | | | | | | | |
| **Outcome at baseline** | 0.61  (0.36 – 0.87) | <.001 | 0.37  (-0.15 – 0.90) | .156 | 0.30  (-0.01 – 0.61) | .058 | 0.16  (-0.50 – 0.81) | .622 | 0.41  (0.07 – 0.76) | .020 | 0.56  (0.03 – 1.08) | .039 |
| **Preference to be alone** | 0.12  (-0.10 – 0.33) | .274 | 0.18  (-0.24 – 0.61) | .381 | -1.74  (-4.30 – 0.82) | .177 | -0.48  (-4.21 – 3.25) | .792 | -1.80  (-4.94 – 1.33) | .251 | 0.24  (-4.34 – 4.82) | .914 |
| Predictor: Pleasantness of being alone | | | | | | | | | | | | |
| **Outcome at baseline** | 0.65  (0.38 – 0.91) | <.001 | 0.43  (-0.09 – 0.95) | .099 | 0.33  (0.00 – 0.65) | .050 | 0.20  (-0.42 – 0.81) | .516 | 0.48  (0.13 – 0.84) | .009 | 0.47  (-0.02 – 0.96) | .058 |
| **Pleasantness of being alone** | 0.04  (-0.18 – 0.26) | .712 | 0.14  (-0.25 – 0.53) | .461 | -0.38  (-2.95 – 2.19) | .764 | -2.09  (-5.37 – 1.18) | .199 | -1.52  (-4.61 – 1.57) | .325 | -3.44  (-7.41 – 0.53) | .086 |

^a^ Results adjusted for age, gender, ethnicity and centre.

^b^ Symptom severity assessed with the Clinical Global Impression Scale.

^c^ Level of functioning assessed with the Global Assessment of Functioning Scale.

^d^ CI, Confidence interval.

Table S15. Clinical Outcomes at 1- and 2-year follow-up predicted by anhedonia, social anhedonia and outcome at baseline – restricted analyses. ^a^

|  | Illness severity ^b^ | | | | | Level of functioning: Symptoms ^c^ | | | | | | | Level of functioning: Disability | | | |
| --- | --- | --- | --- | --- | --- | --- | --- | --- | --- | --- | --- | --- | --- | --- | --- | --- |
|  | 1-year follow-up *(N=42)* | | 2-year follow-up *(N=32)* | | | | 1-year follow-up *(N=46)* | | | 2-year follow-up *(N=31)* | | | 1-year follow-up *(N=46)* | | 2-year follow-up *(N=31)* | |
|  | *b* (CI ^d^) | *p* | *b* (CI) | *p* | | *b* (CI) | | *p* | | *b* (CI) | *p* | | *b* (CI) | *p* | *b* (CI) | *p* |
|  |  |  |  |  | |  | |  | |  |  | |  |  |  |  |
| Predictor: Anhedonia | | | | | | | | | | | | | | | | |
| **Outcome at baseline** | 0.60  (0.34 – 0.86) | <.001 | 0.35  (-0.23 – 0.93) | | .230 | | 0.30  (-0.01 – 0.61) | | .057 | 0.13  (-0.50 – 0.77) | | .674 | 0.42  (0.09 – 0.74) | .014 | 0.56  (0.05 – 1.08) | .033 |
| **Anhedonia** | -0.19  (-0.55 – 0.17) | .294 | -0.19  (-0.84 – 0.46) | | .554 | | 3.08  (-0.89 – 7.05) | | .124 | -0.30  (-5.29 – 4.68) | | .901 | 5.35  (0.74 – 9.97) | .024 | -1.39  (-7.54 – 4.76) | .644 |
|  | | | | | | | | | | | | | | | | |
| Predictor: Social anhedonia | | | | | | | | | | | | | | | | |
| **Outcome at baseline** | 0.60  (0.34 – 0.85) | <.001 | 0.29  (-0.27- 0.85) | | .301 | | 0.29  (-0.01 – 0.59) | | .059 | 0.14  (-0.49 – 0.78) | | .642 | 0.39  (0.07 – 0.71) | .018 | 0.54  (0.02 – 1.06) | .043 |
| **Social anhedonia** | -0.22  (-0.57 – 0.14) | .220 | -0.38  (-1.05 – 0.29) | | .248 | | 3.92  (0.00 – 7.85) | | .050 | 0.59  (-4.87 – 6.05) | | .824 | 6.18  (1.60 - 10.75) | .010 | 0.69  (-6.14 – 7.52) | .836 |

^a^ Results adjusted for age, gender, ethnicity and centre.

^b^ Symptom severity assessed with the Clinical Global Impression Scale.

^c^ Level of functioning assessed with the Global Assessment of Functioning Scale.

^d^ CI, Confidence interval.
